# Li_7_La_3_Zr_2_O_12_/Polymethacrylate-Based Composite Electrolyte with Hybrid Solid Electrolyte Interphase for Ultra-stable Solid-State Lithium Batteries

**DOI:** 10.1007/s40820-025-02041-3

**Published:** 2026-01-12

**Authors:** Tao Li, Guohao Zhao, Zhiyi Zhao, Yaqi Xu, Tianli Wu, Dong-Liang Peng, Qingshui Xie, Ying Xu

**Affiliations:** 1 School of Materials and Energy, Lanzhou University, Lanzhou, 730000 People’s Republic of China; 2 Henan Key Laboratory of Quantum Materials and Quantum Energy, School of Future Technology, Henan University, Kaifeng, 475004 People’s Republic of China; 3 College of Materials, Xiamen University, Xiamen, 361005 People’s Republic of China

**Keywords:** LLZTO, Ionic conductivity, Li_3_N-LiF hybrid SEI, Stable interphase, Solid-state Li batteries

## Abstract

Li_7_La_3_Zr_2_O_12_-based electrolytes have got great promise for solid-state lithium (Li) metal batteries because of their high elastic modulus and wide electrochemical stability window. However, the insufficient contact and heterogeneous Li deposition severely hinder their practical applications. Here, a flexible ternary polymethacrylate (PMA) matrix is designed to incorporate with Ta-doped Li_7_La_3_Zr_2_O_12_ (LLZTO-PMA). The PMA matrix ensures excellent interfacial contact, while the synergistic effects of its polar carbonyl groups and its interaction with LLZTO creating fast interfacial Li^+^ pathways yield a high ionic conductivity of 0.266 mS cm ^−^ ^1^ at 20 °C. Moreover, the interaction between LLZTO and PMA matrix further guides the formation of a hybrid LiF/Li_3_N-rich solid electrolyte interphase, which allows a fast Li^+^ interfacial kinetic due to its lowered Li^+^ diffusion barrier. Consequently, the LLZTO-PMA electrolyte contributes an ultra-stable Li anode interphase, attaining a lifespan exceeding 10,000 h in symmetric cells and retaining over 96% capacity after 600 cycles in full battery, demonstrating a breakthrough for high-performance solid-state batteries.

**Supplementary Information:**

The online version contains supplementary material available at 10.1007/s40820-025-02041-3.

## Highlights

- A molecular engineering of Ta-doped Li_7_La_3_Zr_2_O_12_ (LLZTO) incorporated with polymethacrylate-based (PMA) copolymer moves beyond simple blending to combine the polar carbonyl groups and interfacial Li⁺ transport pathways, yielding high ionic conductivity (0.266 mS cm ^−^ ^1^) and high Li^+^ transference number (0.621) at 20 °C.
- The integration of LLZTO triggers the in situ formation of a hybrid LiF-Li_3_N-rich solid electrolyte interphase with a low Li^+^ diffusion barrier for uniform Li deposition and exceptional interfacial stability.
- The LLZTO-PMA contributes an ultra-stable anode interphase, thus delivering symmetric cell over 10,000 h.

**Supplementary Information:**

The online version contains supplementary material available at 10.1007/s40820-025-02041-3.

## Graphical abstract

**Figure:**
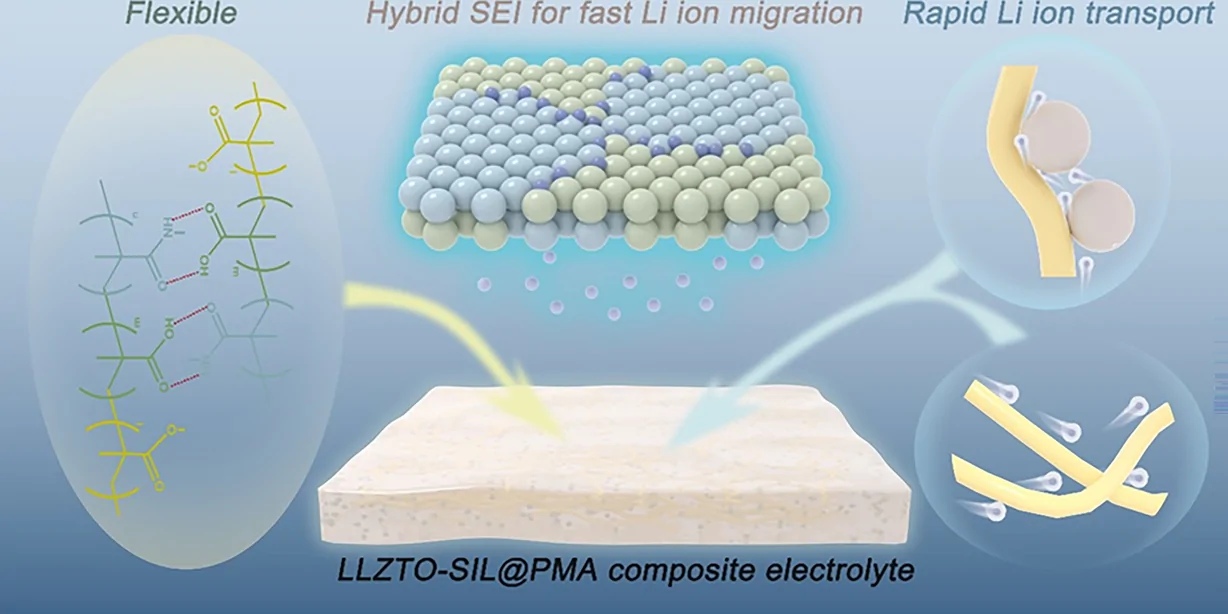

## Introduction

Solid-state Li metal batteries have attracted great attention owing to their non-flammable and high specific capacity properties, particularly in response to the growing pursuit of high energy density and high safety for next-generation batteries [1–3]. Solid-state electrolyte serves as a critical component in such systems, where the Li_7_La_3_Zr_2_O_12_-based (LLZO) electrolytes have been regarded as one of the most promising candidates, owing to their high shear modulus and wide electrochemical stability window [4, 5]. However, the poor interfacial contact impedes Li ion transport, leading to high interfacial impedance and uneven Li deposition [6–8]. Compositing with solid polymer electrolytes (SPEs) or constructing asymmetric electrolytes by merging LLZO into SPEs layers [9, 10] is the common strategy to address the interfacial issues, where the flexible polymer components enhance the interfacial contacts and adapt to the volume changes of electrodes during batteries operation. Therefore, numerous SPEs for constructing LLZO-polymer composite electrolytes have been employed, such as polyethylene oxide [11], poly(vinylidene fluoride-*co*-hexafluoropropylene) [12], polyacrylonitrile [13], 1,3-dioxolane [14], and poly(ethylene glycol) diacrylate [15].

While this approach is promising, the state of the art remains constrained by fundamental compromises. Commonly used SPEs, such as polyethylene oxide, suffer from low ionic conductivity at room temperature [10, 16, 17], while others like polyacrylonitrile exhibit continuous decomposition at the lithium anode, leading to an unstable interface [13, 18, 19]. Furthermore, while ideal solid electrolyte interphase (SEI) components like LiF and Li_3_N are known for their high mechanical strength and ability to suppress dendrites [20, 21], their inherently low ionic conductivity and high Li⁺ diffusion barriers can paradoxically lead to space-charge effects and promote dendrite growth [8, 22, 23]. Therefore, the critical challenge lies in developing a composite electrolyte that simultaneously ensures mechanical robustness, high ionic conductivity, and an interfacial layer that is both stable and highly ion-conductive.

Poly(methyl methacrylate) (PMMA) SPEs demonstrate promise in this regard, as its strong polar carbonyl groups facilitate Li salt dissociation, yielding high ionic conductivity, while also offering favorable interfacial stability with Li anode [24, 25]. However, the poor mechanical strength and film brittleness limit its standalone application, which can hardly restrain Li dendrite penetration and may promote the Li dendrite growth due to the uneven Li^+^ distribution, while the general mitigation strategies include copolymerization, cross-linking, and incorporation with a scaffold [24, 26, 27].

**Fig. 1:**
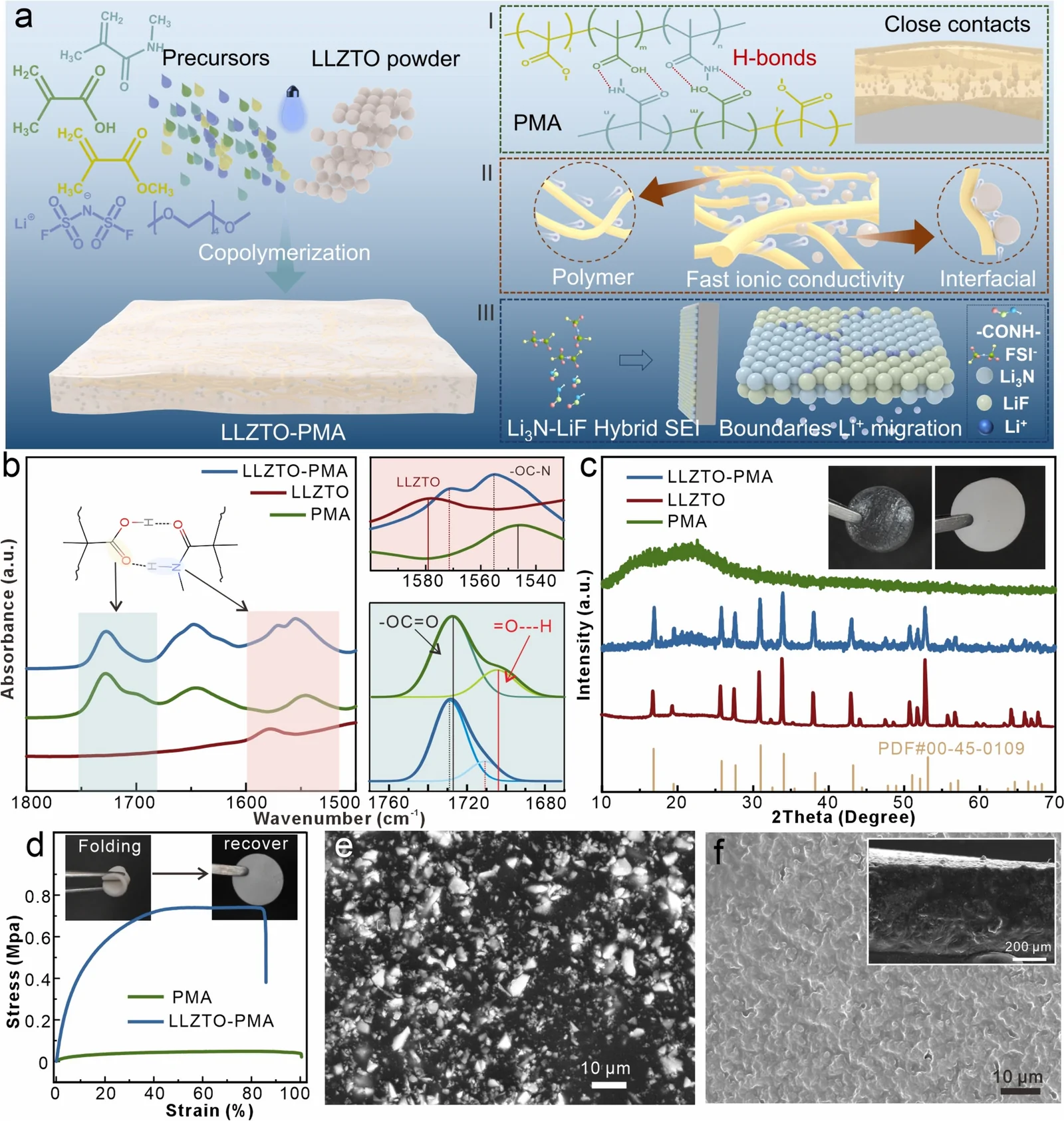
**a** Schematic of synthesis, composition, ion transport, and interphase stabilization mechanism of LLZTO-PMA. **b** FTIR spectra, **c** XRD diffraction patterns, and **d** strain–stress curves of LLZTO, PMA, and LLZTO-PMA, the insets are optical photographs of PMA and LLZTO-PMA films. **e** SEM and **f** cross-sectional SEM images of LLZTO-PMA

Herein, we report a paradigm that moves beyond simple physical blending by demonstrating molecular-level interfacial engineering in a LLZO-based composite electrolyte. We rationally integrate the Ta-doped Li_7_La_3_Zr_2_O_12_ particles with polymethacrylate-based (PMA) SPEs (named as LLZTO-PMA) through an ultraviolet-initiated copolymerization among methacrylic acid (MAA), methyl methacrylate (MMA), and N-methyl methacrylamide (NMMA) (Fig. 1a). This unique PMA matrix firstly provides mechanical toughness through an internal hydrogen-bonding network among carboxyl and amide groups, which enhances the interface contacts and volume change adaptability at the Li anode (Fig. 1a(Ⅰ)). Secondly, its polar carbonyl groups deliver high ionic conductivity, while the interfacial pathways with LLZTO facilitate selective Li ions migration, together enabling the LLZTO-PMA electrolyte to achieve high ionic conductivity of 0.266 mS cm^−1^ with Li ion transference number of 0.621 at 20 °C (Fig. 1a(II)). Moreover, this PMA matrix also acts as a precursor for forming a superior SEI. We reveal that the LLZTO–PMA interface preferentially reduces the lowest unoccupied molecular orbital (LUMO) energy level of both FSI^−^ anion and NMMA, leading to the in situ formation of a hybrid LiF-Li_3_N-rich SEI. Theoretical calculations confirm that this hybrid SEI establishes a dual-phase ion transport pathway with an ultralow Li⁺ diffusion barrier (0.58 eV), significantly lower than that of bare LiF or Li_3_N, thus guiding uniform Li deposition (Fig. 1a(III)). This molecular-level design results in the LLZTO-PMA composite electrolyte that attains exceptional electrochemical performance, including unprecedented stability in Li symmetric cells (> 10,000 h) and high-capacity retention in full cells, thereby addressing the core interfacial challenges in solid-state Li metal batteries.

## Experimental Section

### Materials

The chemicals and materials utilized in this study include N-methyl methacrylamide (NMMAm) (Aladdin, 98.0%), methacrylic acid (MAAc) (Aladdin, > 99.0%), methyl methacrylate (MMA) (Aladdin, 99.0%), tetraglyme (Aladdin, ≥ 99%), tetraglyme (Aladdin, ≥ 99%), 2-hydroxy-2-methyl-1-phenylpropan-1-one (HMPP) (Aladdin, ≥ 97%), lithium bis(fluorosulfonyl)imide (LiFSI) (99.5%), N-methylpyrrolidone (NMP) (99.5%, water content ≤ 50 ppm, Macklin), Ta-doped Li_7_La_3_Zr_2_O_12_ (Li_6.4_La_2_Zr_1.4_Ta_0.6_O_12_) powders were purchased from Wuxi Kai-Star Electro-Optical Materials Co., Ltd; Li foil (battery level), carbon black (Super-P), aluminum foils, and LiFePO_4_ (battery level) were purchased from Guangdong Canrd New Energy Technology Co., Ltd.

### Preparation of LLZTO Pellets, PMA, and GO/HCPA Nanocomposite Papers

#### Synthesis of LLZTO Pellets

The purchased LLZTO powders were compressed under an isostatic pressure of 300 MPa to form pellets. These pellets underwent sintering at 1180 °C for 6 h, with the sintering process being aided by the presence of mother powder. The resulting LLZTO pellets were collected and subjected to sanding using sandpapers of varying mesh sizes to prepare them for further applications.

#### Preparations of PMA

Firstly, LiFSI (3.74 g) was dissolved in tetraglyme (5 mL) at the room temperature, followed by mixing MAAc (0.387 g), MMA (0.9 g), NMMAm (0.446 g), and HMPP (5.0 μL) with stirring. The PMA was subsequently synthesized via copolymerization under 365 nm UV irradiation for 1 h at room temperature.

#### Fabrications of LLZTO-PMA Electrolyte

0, 20, 40, and 60 wt% of LLZTO powders were mixed with the above PMA precursor solution at room temperature, and then, the LLZTO-PMA precursors were dropped into the mold for copolymerization under 365 nm UV irradiation for 1 h at room temperature. To fabricate electrolyte with consistent thickness, a predetermined mass of the electrolyte slurry (PMA precursor solution with LLZTO particles or bare PMA precursor solution) is cast into a glass mold with a fixed area. Since the density of the slurry is consistent, the final thickness is directly and accurately determined by the mass of the slurry poured into the fixed-area mold. This method ensures excellent reproducibility in membrane thickness across different batches.

## Results and Discussion

### Design Principle and Structural Characterizations

The synthesis of LLZTO-PMA is based on the previous publications (Fig. 1a) [28, 29]. To explore the optimal LLZTO content, PMA with various LLZTO mass fractions (0, 20, 40, and 60 wt%) is prepared and tested, which exhibits the highest ionic conductivity with 20 wt% of LLZTO (Fig. S1), thus being selected as the target product and for the overall subsequent characterizations. The thermogravimetric curves comparison also verifies the 20 wt% content of LLZTO in LLZTO-PMA (Fig. S2). Meanwhile, compared to bare PMA, LLZTO-PMA exhibits an enhanced thermal stability (Fig. S2), suggesting an interaction between LLZTO and PMA. Additionally, to optimize the LLZTO-PMA electrolyte thickness, 100–600 μm of LLZTO-PMA electrolytes is prepared and evaluated their critical current densities (Fig. S3). The results show that LLZTO-PMA thinner than ~ 500 μm exhibits reduced critical current densities due to insufficient mechanical strength against Li dendrite penetration, while thicker membranes show increased polarization from higher ionic resistance. Thus, ~ 500 μm is identified as the optimal thickness, balancing mechanical integrity and ionic conductivity for stable cycling. Proton nuclear magnetic resonance (^1^H-NMR) spectroscopies (Fig. S4), Fourier-transform infrared (FTIR) spectroscopies (Fig. S5), and the corresponding optical photographs (Fig. S6) indicate the successful copolymerization of PMA, which are consistent with the previous publications [29]. Additionally, the PMA and LLZTO-PMA display similar characteristic peaks, implying the negligible influence of LLZTO incorporation on the PMA copolymerization. However, the peak at 1580 cm^−1^ that comes from LLZTO exhibits obvious shift after PMA incorporation, indicating an interaction in between (Fig. 1b), which is consistent with the enhanced thermal stability (Fig. S2). These interactions should be attributed to the Li_2_CO_3_ contaminants on LLZTO particle surfaces [30, 31], which show interactions with carboxyl and amide groups in PMA [32, 33]. These interactions as well as the physically blending of LLZTO particles with PMA disrupt the hydrogen bonds between carboxyl and amide groups in PMA, as evidenced by an intensity reduction of the hydrogen-bond signature at ~ 1710 cm^−1^ and obvious peak shifts of –OC–N and –OC = O from 1547 and 1726 cm^−1^ to 1555 and 1729 cm^−1^, respectively (Fig. 1b) [28, 29], as well as the decreased intensity and shifted characteristic peak of hydrogen bond at ~ 3548 cm^−1^ (Fig. S5). This disruption ultimately exposes free amide and carboxyl functional groups in the LLZTO-PMA.

The incorporation of LLZTO transforms the color of originally PMA film from transparent to milky white (insets in Fig. 1c). X-ray diffraction (XRD) spectra reveal that the LLZTO remains its original crystalline (Fig. 1c), matching well with the standard cubic-LLZO (PDF No. 00-45-0109). The broad amorphous hump at around 20°–25° is attributed to the PMA SPEs. According to the strain–stress curves (Fig. 1d), both PMA and LLZTO-PMA films exhibit superior elasticity with tensile strains exceeding 80%, while the LLZTO incorporation enhances the mechanical strength from 48 Kpa (PMA) to 740 Kpa (LLZTO-PMA). Additionally, the optical images of bending/folding LLZTO-PMA film also suggest its flexibility without structural failure (insets in Fig. 1d), further demonstrating its superior mechanical performance. According to the scanning electron microscope (SEM) images and the corresponding elemental mappings, the bare PMA film with thickness of ~ 500 μm displays porous structure (Fig. S7), which is resulted from the volumetric shrinkage during copolymerization. In contrast, the LLZTO-PMA film presents smooth and pore-free morphology with thickness of ~ 500 μm, where the LLZTO particles are dispersed and encapsulated within PMA (Figs. 1f and S8). The pore-free morphology is attributed to the interaction of LLZTO and PMA, which reduces the bulk shrinkage during copolymerization. For comparison, bare LLZTO pellet with around 500 μm thickness (Fig. S7c, d) is also prepared for the following electrochemical tests.

### Electrochemical Characterizations and Mechanism Investigations

**Fig. 2:**
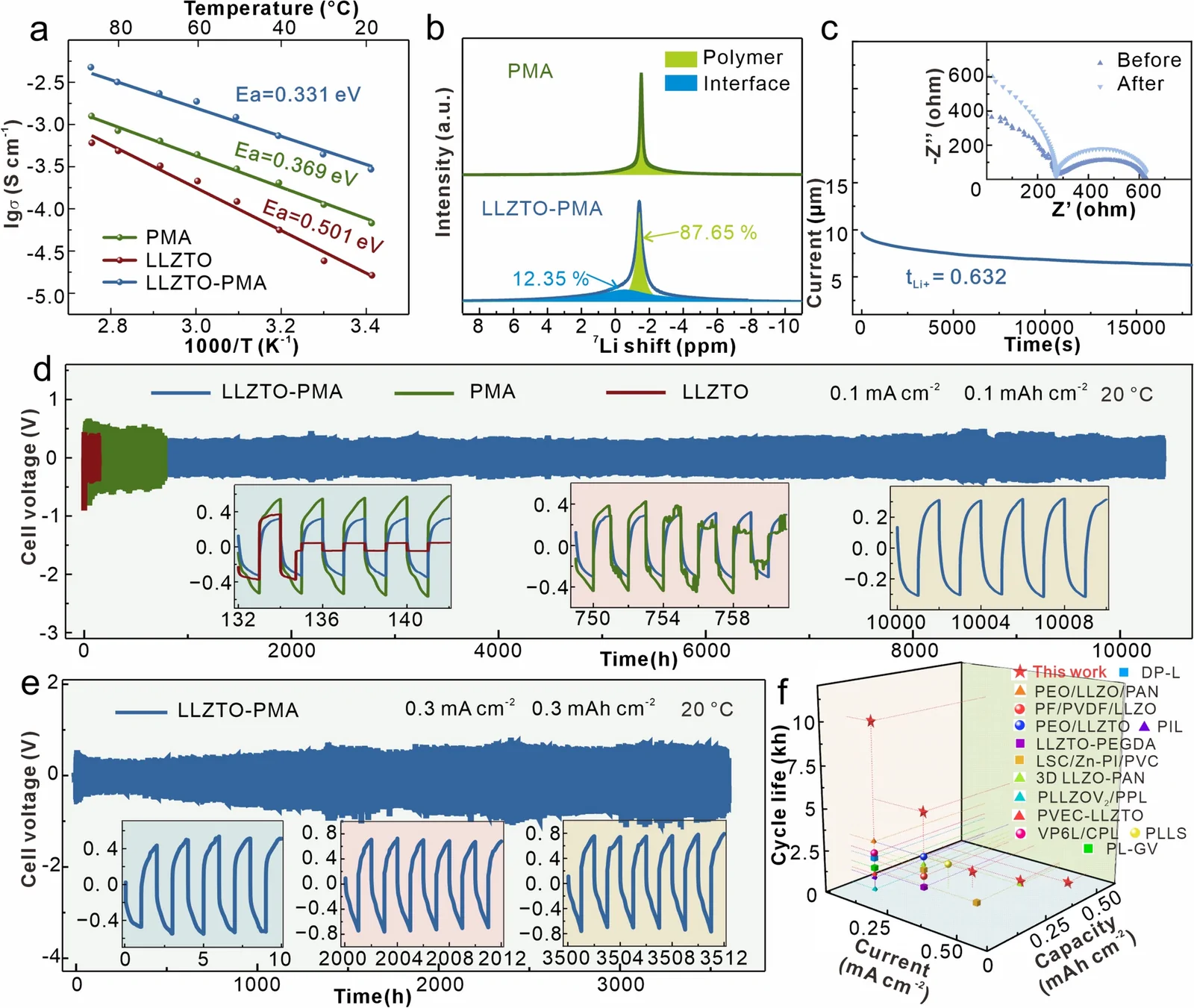
**a** Arrhenius plots and activation energies of LLZTO-PMA, PMA, and LLZTO electrolytes. **b** ^7^Li-NMR spectroscopies of LLZTO-PMA and PMA electrolytes. **c** Chronoamperometry curve and AC impedance spectra before and after polarization of Li|LLZTO-PMA|Li cell. Galvanostatic Li plating/stripping curves of Li||Li cells with LLZTO-PMA, PMA, and LLZTO electrolytes at 20 °C with **d** 0.1 mA cm^−2^/0.1 mAh cm^−2^, and **e** 0.3 mA cm^−2^/0.3 mAh cm^−2^ (the insets are detailed voltage profile comparisons). **f** Comparisons with other previously reported works for LLZO-based composite electrolytes [14, 33, 38–48]

The ionic conductivities at different temperatures of LLZTO, PMA, and LLZTO-PMA electrolytes are measured from 20 to 90 °C (Fig. S9). The corresponding Arrhenius plots reveal that the LLZTO-PMA film displays the highest ionic conductivity of 0.266 mS cm^−1^, in comparison with bare LLZTO of 0.016 mS cm^−1^ and PMA of 0.07 mS m^−1^ at 20 °C (Fig. 2a). Meanwhile, LLZTO-PMA electrolyte exhibits an activation energy of 0.331 eV, which is lower than both bare LLZTO (0.369 eV) and PMA (0.501 eV), suggesting an enhanced kinetics in Li ion transport. To investigate the Li ion transport mechanism of LLZTO-PMA, solid-state ^7^Li nuclear magnetic resonance spectroscopies are conducted, which discovers an additional interfacial transport pathway in LLZTO-PMA electrolyte (Fig. 2b), in comparison with the conventional Li ion migration pathway through the polymer matrixes in PMA. According to the further quantitative analysis, the interfacial transport contributes 12.35% to the overall ionic conductivity, while the polymer bulk transport accounts for the remaining 87.65%. This additional interfacial transport pathway contributes to the enhanced ionic conductivity in LLZTO-PMA [34, 35]. Meanwhile, the LLZTO-PMA electrolyte also demonstrates an increased Li ion transference number (*t*_Li+_ = 0.632) (Fig. 2c) compared to PMA (*t*_Li+_ = 0.435) and bare LLZTO (*t*_Li+_ = 0.520) electrolytes (Fig. S10). This enhanced Li ion transference number should be attributed to the LLZTO phase, which is rich in Lewis acid sites. These sites interact with the anions from the Li salt, thus anchoring or slowing down anion mobility and consequently increasing the Li ion transference number. Due to the rapid Li ion transport kinetics and continuous interfacial contact, LLZTO-PMA electrolyte attains an enhanced critical current density of 0.8 mA cm^−2^ at 20 °C, while the bare LLZTO and PMA electrolytes only operate at 0.25 and 0.35 mA cm^−2^, respectively (Fig. S11), demonstrating an improved capability in suppression of Li dendrite growth. Moreover, the LLZTO-PMA electrolyte also displays a broadened electrochemical stability window of 4.76 V in comparison with 4.2 V of bare LLZTO and 4.26 V of PMA (Fig. S12). This enhanced voltage window may be attributed to the exposed amide functional groups in the LLZTO-PMA that may generate a stable cathode electrolyte interphase layer [36, 37].

The long-term cycling stability of electrolytes to Li anode is measured through the Li||Li symmetric cells at 20 °C. Li|LLZTO-PMA|Li cell delivers over 10,000 h with overpotential of ~ 400 mV at the 0.1 mA cm^−2^/0.1 mAh cm^−2^ (Fig. 2d), while Li|LLZTO|Li and Li|PMA|Li cells only work for 140 and 740 h with ~ 420 and ~ 680 mV overpotentials, respectively. The detailed voltage profiles confirm the short-circuit state in both Li|LLZTO|Li and Li|PMA|Li cells, while demonstrating the stable and low-overpotential operation of Li|LLZTO-PMA|Li cell (insets in Fig. 2d). At a current density of 0.2 mA cm^−2^, the bare Li|LLZTO|Li cell can hardly work and the Li|PMA|Li cell only survives ~ 630 h with ~ 750 mV overpotential. In contrast, Li|LLZTO-PMA|Li cell operates stably over 4700 h with overpotential of ~ 400 mV (Fig. S13). Moreover, Li|PMA|Li cell can hardly work at 0.3 mA cm^−2^, but Li|LLZTO-PMA|Li operates over 3500 h with ~ 480 mV overpotential (Fig. 2e) and even maintains stable operations at both 0.4 and 0.5 mA cm^−2^ (maintaining stable operation for over 750 h with voltage polarization of approximately 800 mV at 0.4 mA cm^−2^ and over 80 h at 0.5 mA cm^−2^, as shown in Figs. S14 and S15). The voltage fluctuations during cycling at different current density should be attributed to the dynamic changes at the electrode–electrolyte interface, the formation and dissolution of the SEI, localized inhomogeneities in ion transport, and sometimes the randomly changed test temperature. These fluctuations are reproducible and do not indicate instability of cells; rather, they reflect intrinsic material behavior and kinetic processes under dynamic cycling conditions. Overall, the LLZTO-PMA electrolyte shows an ultrastability to Li anode, which is superior to most similar previous works (Fig. 2f) [14, 33, 38–48].

**Fig. 3:**
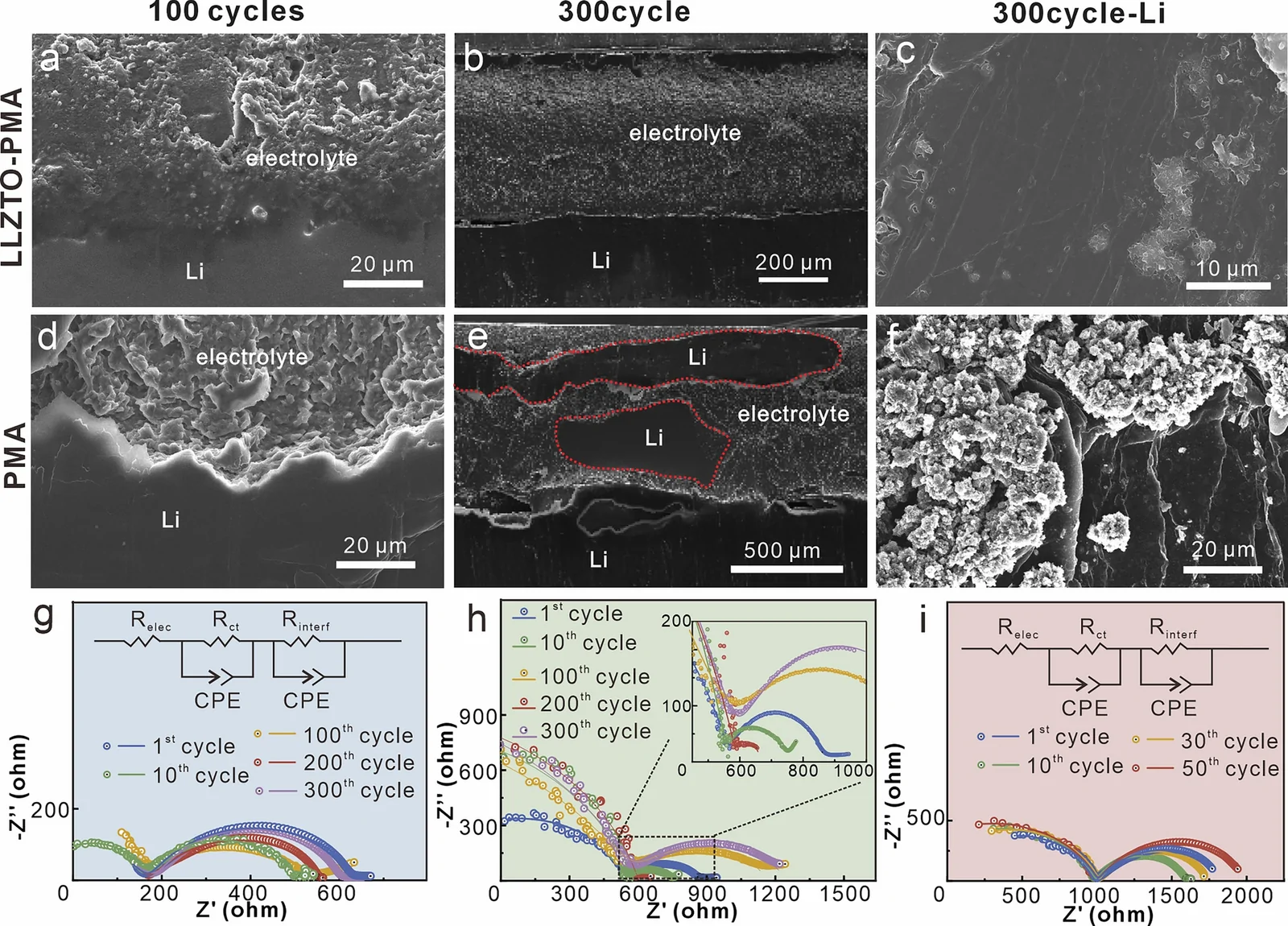
Cross-sectional SEM images of Li-electrolyte and surface SEM images of Li anode in symmetric cells with **a**–**c** LLZTO-PMA and **d**–**f** PMA electrolytes after 100 and 300 cycles with 0.1 mA cm^−2^ at 20 °C. Nyquist plots of symmetric cells with **g** LLZTO-PMA, **h** PMA, **i** LLZTO electrolytes at 20 °C after 1, 10, 100, 200, and 300 cycles with 0.1 mA cm^−2^ (the LLZTO electrolytes are only tested after 1, 10, 30, and 50 cycles due to its limited cycling stability; the insets are equivalent circuit diagrams or magnified area)

To investigate the reasons for the ultra-stable LLZTO-PMA-based cells operation, symmetric cells with various electrolytes after different cycles at 0.1 mA cm^−2^ are disassembled for interfacial characterizations. In comparison with the severe interfacial delamination of LLZTO with Li anode after 50 cycles (Fig. S16c), cross-sectional SEM images reveal that both PMA and LLZTO-PMA electrolytes retain close contact with Li anodes after cycling (Figs. 3a–e, S16, and S17), demonstrating the superior performance of the PMA for adapting the volume changes of electrodes during cell operations. However, LLZTO-PMA exhibits straight interfacial boundary with uniform Li deposition (Figs. 3a, S16a, and S17a), while PMA exhibits an arched interfacial boundary with Li anode (Figs. 3d, S16b, and S17b), implying a heterogeneous Li deposition. This obvious distinction demonstrates that LLZTO-PMA can effectively restrain Li dendrite growth but the bare PMA although enabling good interfacial contact with Li anode, its inability to suppress heterogeneous Li deposition governed by multiple factors. Furthermore, metallic Li penetrations are observed in the PMA bulk (Fig. 3e), where the highlight areas can hardly detect the characteristic elemental signals of PMA by energy-dispersive X-ray spectroscopy (EDX) (Fig. S18a), in comparison with the intact LLZO-PMA after 300 cycles (Figs. 3b and S18b). The observed Li permeation reveals that PMA lacks the mechanical robustness required to block dendrite piercing. Additionally, the Li anode surface images also verify the uniform Li deposition in Li|LLZTO-PMA|Li cell in comparison with mossy Li deposition in Li|PMA|Li cell (Fig. 3c, f).

The stable interphase between LLZTO-PMA electrolyte and Li anode after cycling is further verified by the electrochemical impedance spectra (EIS) characterizations. The calculated interfacial impedances based on equivalent circuit are shown in Table S1. Specifically, Li|LLZTO-PMA|Li cell presents an interfacial impedance of 472 Ω after the first cycle, which decreases to 354 Ω (10 cycles) and increases to 365 Ω (100 cycles), followed with maintaining at ~ 400 Ω after 200 and 300 cycles (Fig. 3g). The maintained interfacial impedance suggests a stable interphase with homogeneous Li deposition [28, 49]. In comparison, Li|PMA|Li cell displays fluctuating interfacial impedances of 310 → 172 → 581 → 51 → 656 Ω after 1, 10, 100, 200, and 300 cycles (Fig. 3h), where the suddenly decreased impedance reveals a soft short circuit like inactive Li penetrations in PMA (Fig. 3e) [50] and the progressively increased impedances indicate an interphase degradation [28, 49]. This degradation is also discovered in Li|LLZTO|Li cell with the increased interfacial impedances of 883 → 616 → 748 → 1040 Ω after 1, 10, 30, and 50 cycles (Fig. 3i). These interfacial impedance variations are consistent with the interphase evolutions observed by SEM.

**Fig. 4:**
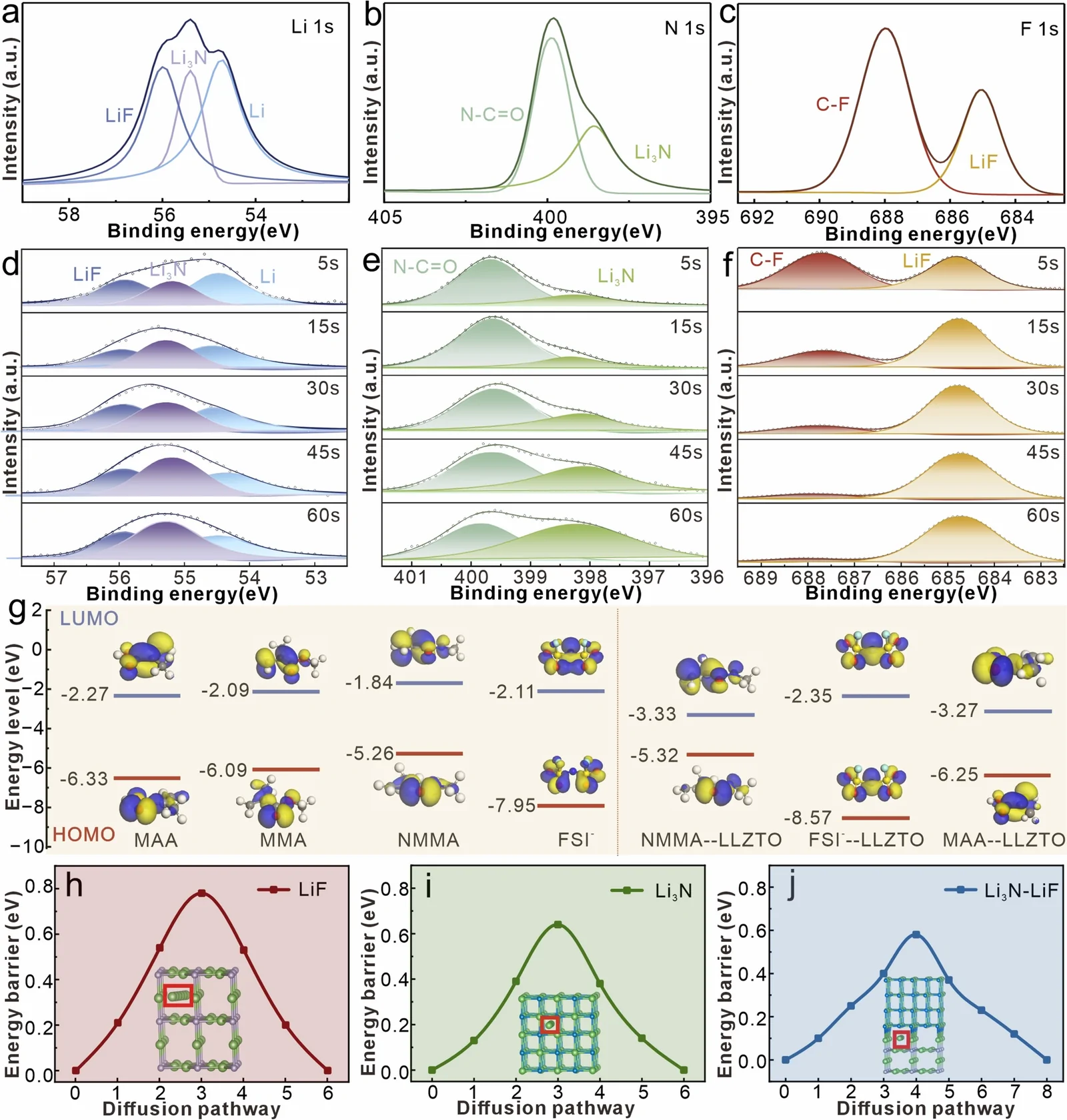
High-resolution XPS spectra and the different etching depths of **a**, **d** Li 1*s*, **b**, **e** N 1*s*, and **c**, **f** F 1*s* on Li surface within Li|LLZTO-PMA|Li cell after 10 cycles at 0.1 mA cm^−2^. **g** HOMO and LUMO energy levels of NMMA, MMA, MAA, and FSI^−^ anion with/without LLZTO. Detailed diffusion barrier and pathways of Li adatom on **h** LiF, **i** Li_3_N, and **j** Li_3_N-LiF bulks

To further explore the root causes for the stable interphase with dendritic-free Li deposition in LLZTO-PMA-based cells, the SEI layers on Li anodes after cycling are analyzed through X-ray photoelectron spectroscopy (XPS) (Fig. S19). The high-resolution Li 1*s* peak on PMA-based Li anode is assigned as LiF (55.9 eV) and Li (54.8 eV) (Fig. S20a) [51]. However, an additional peak at 55.4 eV, corresponding to Li_3_N [51], is observed on LLZTO-PMA-based Li anode (Fig. 4a). Furthermore, the N 1*s* peak on PMA-based Li anode is primarily from amide group (399.9 eV) (Fig. S20b), being attributed to the residual PMA electrolyte, while the N 1*s* peak on LLZTO-PMA-based Li anode is assigned to both amide group and Li_3_N (398.3 eV) [21] (Fig. 4b). The formation of Li_3_N is attributed to the reactions between Li anode and free amide groups in LLZTO-PMA electrolyte. Meanwhile, both F 1*s* peaks (Figs. S20c and 4c) and Li 1*s* peaks (Figs. S20a and 4a) indicate that an increased LiF content on LLZTO-PMA-based Li anode, which may be ascribed to that the LLZTO induces the decomposition of FSI^−^ anions in PMA matrixes. Additionally, XPS depth profiling further demonstrates the coexistence of both Li_3_N and LiF components, where the Li_3_N content increases with the depth etching (Fig. 4d, e), while the LiF almost maintains constant (Fig. 4d, f). This Li_3_N enrichment in the inner SEI near the Li anode may originate from the preferential interfacial reactions between exposed amide groups in LLZTO-PMA and metallic Li anode. Overall, the SEI layer on LLZTO-PMA-based Li anode is rich in both Li_3_N and LiF, which have been demonstrated to restrain Li dendrite growth due to their inherent mechanical strength and ionic conductivity [20, 21].

To further investigate the fundamental mechanism for the Li_3_N-LiF SEI formation, the highest occupied molecular orbital (HOMO) and lowest unoccupied molecular orbital (LUMO) energy levels of NMMA, MMA, MAA monomers, and FSI^−^ anion with/without LLZTO are calculated systematically [52]. In the bare PMA, MAA exhibits the lowest LUMO energy (− 2.27 eV) (Fig. 4g). However, the hydrogen bonds between MAA and NMMA restrict the exposure of MAA functional groups, thus suppressing its preferential decomposition on Li anode. This inhibition enables FSI^−^ anions with the second lowest LUMO (− 2.11 eV) to dominantly decompose into LiF component on Li anode [53, 54], which is consistent with the above LiF-rich SEI on PMA-based Li anode. In comparison, the incorporation of LLZTO disrupts hydrogen-bond interactions within the PMA matrixes, exposing reactive amide groups from NMMA and carboxyl groups from MAA, that actively participate in SEI formation. Furthermore, the density functional theory (DFT) calculations reveal an energy hierarchy inversion, where the LUMO energy of NMMA (decreasing from − 1.84 to − 3.33 eV) and MAA (decreasing from − 2.27 to − 3.25 eV) is lower than FSI^−^ (decreasing from − 2.11 to − 2.35 eV) (right side of Figs. 4g and S21), thus generating Li_3_N-rich phase as the primary reduction site through preferential amide groups decompositions [53, 54]. Besides, the decomposition of MAA introduces organic components into the SEI, acting as a binding matrix, which enhances the structural integrity and cohesion of the SEI. Concurrently, the FSI^−^ anions with reduced LUMO energy contribute complementary LiF formation, ultimately creating a hybrid SEI layer rich in both Li_3_N and LiF. Meanwhile, the enhanced FSI^−^ anions decompositions also result in an increased LiF component on LLZTO-PMA-based Li anode. Furthermore, the HOMO analysis shows the highest HOMO energy of NMMA (− 5.32 eV) with LLZTO incorporation, which triggers a preferential oxidation at the cathode, forming a stable cathode electrolyte interphase layer and expanding the electrochemical stability window of LLZTO-PMA-based batteries [53, 54].

Additionally, according to the DFT calculations, the diffusion barrier of Li ion is 0.78 eV and is 0.64 eV in the bulk phases of pure LiF and Li_3_N layer, which are higher than that in the Li_3_N-LiF hybrid bulk phase (0.58 eV) (Fig. 4h-j). The reduced diffusion barrier is attributed to that the Li ions preferentially migrate through the grain boundaries between Li_3_N and LiF [55]. Therefore, compared to the PMA-based Li anode surface that only rich in LiF, the LLZO-PMA-based Li anode surface, enriched with Li_3_N-LiF, exhibits a lower Li ion diffusion barrier. This enhanced ion diffusion kinetics is consistent with the lower interfacial impedance observed in the LLZTO-PMA-based symmetric Li cells compared to bare PMA-based cells after cycling (Fig. 3g, h), which effectively facilitates rapid Li ion transport at the electrolyte–Li anode interphase, thereby suppressing dendrite growth induced by the space-charge layer while minimizing interfacial electrochemical polarization [55–57]. Consequently, compared to PMA, the LLZO-PMA-based Li anode demonstrates a more uniform and denser Li deposition, ultimately improving the overall battery performance.

### Batteries Performance Characterizations

**Fig. 5:**
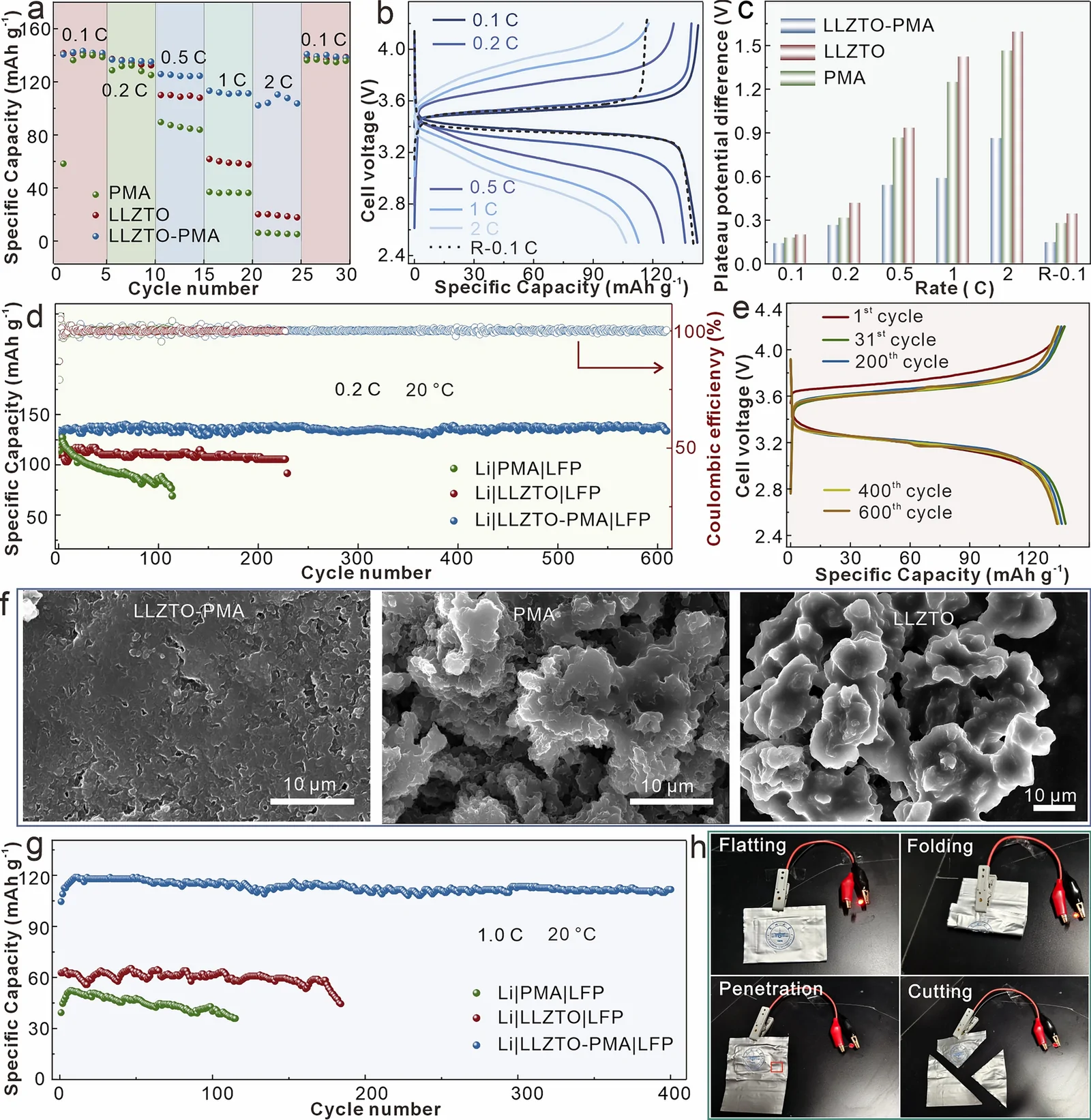
**a** Rate capabilities and **b** the corresponding charge–discharge curves of Li|LLZTO-PMA|LFP at different rates, **c** voltage gap between charge and discharge plateaus at different rates, **d** cycling performance at 0.2 C and **e** their voltage profiles at different cycles, **f** SEM images of Li anode after 100 cycles at 0.2 C, and **g** cycling performance at 1.0 C for Li||LFP batteries with LZLTO, PMA, and LLZTO-PMA electrolytes. **h** Optical images of Li|LLZTO-PMA|LFP pouch cell lighting up light-emitting diode at the abuse conditions

Based on the superior electrochemical performance, full batteries with LiFePO_4_ (LFP) cathodes, Li foil anodes, and the corresponding electrolytes are assembled and tested at 20 °C. The Li|LLZTO-PMA|LFP battery exhibits enhanced rate capability and reversibility (Fig. 5a and 5b), which delivers 143.79, 136.29, 125.38, 112.89, and 101.90 mAh g^−1^ discharge-specific capacity at the 0.1, 0.2, 0.5, 1.0, and 2.0 C, respectively (1.0 C is defined as a current density of 170 mA g^−1^, which is based on the theoretical specific capacity of LFP (170 mAh g^−1^)), and recovers to 140.39 mAh g^−1^ at the reversed 0.1 C with a recovery of 97.63%. In contrast, LLZTO- and PMA-based batteries display lower capacities. Voltage profile comparisons reveal that the Li|LLZTO-PMA|LFP enables higher discharge plateaus and lower charge plateaus at all rates (Fig. S22), further demonstrating a reduced electrochemical polarization (Fig. 5c) and contributing its enhanced rate capability. Moreover, Li|LLZTO-PMA|LFP battery delivers a discharge-specific capacity of 134.13 mAh g^−1^ at the initial cycle at 0.2 C, which attains 138.16 mAh g^−1^ after activation with a capacity retention ratio of 96.79% after 610 cycles (Fig. 5d). The average Coulombic efficiency is 99.89% of Li|LLZTO-PMA|LFP battery, while the nearly overlapped charge–discharge curves at different cycles further confirm its exceptional cycle stability (Fig. 5e). In comparison, the initial capacity of Li|PMA|LFP and Li|LLZTO|LFP is 114.09 and 110.22 mAh g^−1^, which sharply drops to 69.01 mAh g^−1^ after 114 cycles and is short-circuited after 227 cycles, respectively. The detailed charge–discharge curves also verify their capacity fading with cycling (Fig. S23). SEM characterizations reveal that the LLZTO-PMA-based Li anode displays uniform Li deposition with a close interfacial contact, in comparison with the mossy Li deposition with discontinuous or uneven interfacial contacts for LLZTO and PMA electrolytes after cycling (Figs. 5f and S24). As the results, the Li|LLZTO-PMA|LFP battery displays lowest interfacial impedance (Fig. S25), which also verifies its stable interphase, thus contributing to the superior cycling performance. Meanwhile, the enhanced interfacial impedance of Li|PMA|LFP than Li|LLZTO|LFP after cycling is ascribed to the accumulations of side reaction products and inactive Li at PMA-Li interface, both of which hinder the transport of Li ions. Additionally, Li|LLZTO-PMA|LFP battery also exhibits a better cycling performance at 1.0 C, which delivers 119.19 mAh g^−1^ after activation and maintains 112.02 mAh g^−1^ after 400 cycles, with a capacity retention ratio of 93.98% (Fig. 5g). However, the Li|PMA|LFP and Li|LLZTO|LFP batteries display lower capacities and rapid capacity decays. The detailed Coulombic efficiency and voltage profiles comparisons further confirm the enhanced performance of Li|LLZTO-PMA|LFP battery (Figs. S26 and S27). Over a comprehensive comparison, the electrochemical performance of LLZTO-PMA, including ionic conductivity, rate capability, full battery performance, remains commendable and positions among the leading systems reported (Table S2).

Moreover, high-voltage NCM811 cathodes are further assembled with Li anodes and LLZTO-PMA, PMA, and LLZTO electrolytes for full batteries testing at 0.5 C and 20 °C (Fig. S28), where the LLZTO-PMA electrolyte also exhibits superior performance. Specifically, the LLZTO-PMA-based battery displays 150 mAh g^−1^ at the initial after activation, which retains approximately 70% of its capacity after 300 cycles at 0.5 C, while both PMA- and LLZTO-based batteries suffers from rapid capacity fade under the same conditions. This enhanced performance is consistent with the higher oxidation potential of LLZTO-PMA electrolyte revealed by LSV measurements (Fig. S12), confirming its compatibility with high-voltage cathodes. Furthermore, Li|LLZTO-PMA|LFP pouch cell is assembled and powers a commercial light-emitting diode (Fig. 5h), which maintains operation after battery abuse tests of folding, penetration, and cutting. It is worth noting that a dimming of the LED light after the cutting tests occurs because the removed portion of the battery (including active materials and electrolyte) no longer contributes to the energy supply, resulting in a reduction in total capacity and energy output. These abuse tests demonstrate the feasibility of LLZTO-PMA electrolyte for the practical applications in high safety, high energy density, and flexible batteries.

## Conclusions

In summary, we have developed a molecularly engineered composite electrolyte by integrating LLZTO particles into a PMA SPE, where the polar carbonyl groups in PMA and the additional interfacial ionic transport pathways between LLZTO and PMA matrixes synergistically enable LLZTO-PMA a high ionic conductivity of 0.266 mS cm^−1^ at 20 °C. The internal hydrogen-bonding network within the PMA enhances mechanical robustness and interfacial adaptability, effectively accommodating volume changes during cycling. More importantly, we demonstrate that the incorporation of LLZTO disrupts the hydrogen-bonding structure of PMA, exposing functional groups that preferentially participate in the formation of a hybrid LiF-Li_3_N-rich SEI. Theoretical calculations confirm that this unique SEI exhibits a low Li⁺ diffusion barrier, thus facilitating uniform Li⁺ flux and suppressing dendrite growth. As a result, the LLZTO-PMA-based Li anode exhibits ultra-stable and homogeneous Li deposition and the corresponding symmetric cells deliver over 10,000 h at 0.1 mA cm^−2^. Moreover, the corresponding Li||LFP battery maintains 133.73 mAh g^−1^ capacity after 610 cycles with a capacity retention over 96% at 0.2 C. This strategic approach of designing composite solid electrolytes through molecular-level interfacial engineering effectively addresses key challenges in ionic conductivity, interfacial stability, and Li deposition behavior, offering new insights into the rational construction of high-performance solid-state Li metal batteries.

## Supplementary Information

Below is the link to the electronic supplementary material.

Supplementary file1 (DOCX 10243 KB)
