# Supplementary material for: Li7La3Zr2O12/Polymethacrylate-Based Composite Electrolyte with Hybrid Solid Electrolyte Interphase for Ultra-stable Solid-State Lithium Batteries
Source: Nanomicro Lett. 2026 Jan 12;18:189. doi: 10.1007/s40820-025-02041-3 (PMC12791096; doi:10.1007/s40820-025-02041-3)
Supplement: Supplementary file 1 — Supplementary file1 (DOCX 10243 KB) [file 40820_2025_2041_MOESM1_ESM.docx]

Supporting Information for

**Li_7_La_3_Zr_2_O_12_/Polymethacrylate-Based Composite Electrolyte with Hybrid Solid Electrolyte Interphase for Ultra-stable Solid-State Lithium Batteries**

Tao Li^1^, Guohao Zhao^1^, Zhiyi Zhao^1^, Yaqi Xu^1^, Tianli Wu^2^*, Dong-Liang Peng^3^, Qingshui Xie^3^*, Ying Xu^1^*

^1^ School of Materials and Energy, Lanzhou University, Lanzhou 730000, P. R. China

^2^ Henan Key Laboratory of Quantum Materials and Quantum Energy, School of Future Technology, Henan University, Kaifeng 475004, P. R. China

^3^ College of Materials, Xiamen University, Xiamen 361005, P. R. China

*Corresponding authors. E-mail: [tianliwu@henu.edu.cn](mailto:tianliwu@henu.edu.cn) (Tianli Wu); [xieqsh@xmu.edu.cn](mailto:xieqsh@xmu.edu.cn) (Qingshui Xie); [xuying@leu.edu.cn](mailto:xuying@leu.edu.cn) (Ying Xu)

**S1 Experimental section**

**S1.1 Materials characterizations**

The X-ray diffraction (XRD) patterns within the 2θ range from 10° to 70° were characterized using an X-ray diffractometer (Rigaku D/max-2400) with Cu Kα radiation (λ = 1.5406 Å). Scanning electron microscopy (SEM, Apreo S with a field emission electron gun FEG) was employed to analyze the morphology and elemental distribution of the electrolyte and lithium anode before and after cycling. The chemical bonds and elemental information of the solid electrolyte interface (SEI) were characterized by X-ray photoelectron spectroscopy (XPS, SHIMADZU Axis Supra) with Ar^+^ ion etching for depth profiles analysis using an Al Kα monochromatic X-ray source. Fourier transform infrared spectroscopy (FT-IR) spectra were observed by THEMOR-FILSHER NEXUS 670 in the range of 4000~400 cm^−1^. The nuclear magnetic resonance (¹H-NMR) spectra were measured on Bruker Avance Neo 600WB spectrometer. The ^7^Li solid-state nuclear magnetic resonance (SSNMR) spectra were measured on Bruker Avance Neo 400WB spectrometer. Thermogravimetric Analysis (TG) was tested in a nitrogen atmosphere with a temperature range of 30 to 600°C and a heating rate of 10°C/min on Horiba LabRAM HR Evolution. The strain-stress curves are obtained on a universal tensile testing machine (Instron 5697).

The samples for SEM and XPS are prepared by dissolving the electrolytes-Li electrode in tetrahydrofuran (THF) at 40°C for 1 hour in an argon-filled glove box, ensuring the Li anode surface remained intact for analysis.

**S1.2 Electrochemical measurements**

All cells/batteries were assembled in the Ar-filled glovebox with O_2_ and H_2_O less than 0.01 ppm. All cells/batteries were tested on the NEWARE battery test system at 20 °C.

***S1.2.1 Interfacial impedance tests***

The interfacial impedances of batteries after different cycles were tested through electrochemical impedance spectroscopy (EIS) on the electrochemical workstation PRINCETON PMC-2000A with frequency range of 1 MHz to 0.01 Hz with a disturbance amplitude of 10 mV.

***S1.2.2 Ionic conductivity tests***

The ionic conductivity of LLZTO, PMA and LLZTO-PMA electrolytes was tested through EIS with frequency range of 1 MHz to 0.01 Hz and between 20 °C and 90 °C. The electrolytes were sandwiched between two stainless-steel disks, and the ionic conductivity (δ) was calculated from formula (S1):

$\delta=\frac{L}{S\times R}$ (S1)

Where the L (cm) is the thickness of the electrolyte film, S (cm^2^) is the electrode area, and R (Ω) is the resistance value of the bulk electrolyte.

The LLZTO electrolyte pellet was first mechanically polished to improve surface smoothness. The polished pellet was then sputter-coated with a gold layer for 100 seconds on both top and bottom surfaces. Finally, it was sandwiched between stainless steel electrodes for EIS measurement under applied pressure of 0.6 MPa. In contrast, the PMA and LLZTO-PMA electrolytes, due to their good contact with the stainless steel electrodes, could be tested directly without any additional surface coating.

***S1.2.3 Li ion transference number tests***

The Li ion transference number of LLZTO, PMA and LLZTO-PMA electrolytes was measured by chronoamperometry and AC impedance spectra on the electrochemical workstation PRINCETON PMC-2000A at 20 °C. The EIS spectra before and after polarization were tested with a frequency range of 1 MHz to 0.01 Hz. The polarization voltage is 50 mV, and the cells were assembled by electrolytes sandwiching between two Li disks. The Li ion transference number is calculated from formula (S2):

$t_{{Li}^{+}}=\frac{I_{ss}(\Delta V-I_{0}R_{0})}{I_{0}(\Delta V-I_{ss}R_{ss})}$ (S2)

Where I_0_, I_ss_, ΔV, R_0_ and R_ss_ are the initial and stead current value, polarization voltage, and interfacial resistances before and after polarization, respectively.

***S1.2.4 Li||Li cells assembly and tests***

The Li||Li cells were assembled by sandwiching electrolytes (LLZTO, PMA and LLZTO-PMA) between two Li foils. The cycling stability is evaluated by conducting galvanostatic charge-discharge cycling tests on the NEWARE battery testing system at 20 °C, using various combinations of current densities and capacities: 0.1-0.5 mA cm^-2^/0.1-0.5 mAh cm^-2^.

***S1.2.5 Critical current density tests***

The critical current densities were measured by galvanostatic cycling of Li||Li cells with LLZTO, PMA and LLZTO-PMA electrolytes, the current density was increased stepwise from 0.05 to 1 mA cm^-^² with increments of 0.05 mA cm^-^² at 20 °C. Li plating/stripping at every step is alternate for 2 hours.

***S1.2.6 Oxidation potential test***

The electrochemical stability window of various electrolytes was obtained through the linear sweep voltammetry (LSV) of Li|electrolyte|SS cells with the scan rate of 5 mV s^-1^ and potential range of 3 to 6.0 V (*vs.* Li^+^/Li) at 20 °C.

***S1.2.7 Li||LFP coin cells assembly and tests***

LFP was mixed with carbon black and PVDF with weight ratio of 8:1:1 in NMP. the slurry was coated onto aluminum foil with an average weight of approximately 2.0 mg·cm^-2^, followed by vacuum drying at 120°C for 6 hours. The aluminum foils loaded with LFP served as the cathodes, while Li foils with 200 μm thickness were used as the anodes. The diameter of the electrode is 1.0 cm with the calculated N/P ratio of approximately 121.2. Finally, the Li||LFP coin cells were assembled by inserting cathode, electrolyte, and Li anode orderly in CR-2032 cell base, followed with a 0.65 Torr press for encapsulation.

The cycling performance of the batteries were evaluated using the NEWARE battery testing system at 20 °C, with a charge-discharge rate of 0.2 C and 1.0 C within a voltage range of 2.5 to 4.2 V (*vs.* Li/Li^+^). The rate capability tests were conducted at 20 °C with various rates of 0.1, 0.2, 0.5, 1.0, and 2.0 C for 5 cycles each, followed by a recovery test at 0.1 C.

The voltage hysteresis plot illustrates the voltage difference (ΔV) between the charging and discharging curves at 50% state of charge (or discharge, indicating the midpoint of the capacity during the charging/discharging process) under the same current density. The calculation formula for voltage hysteresis is as follows:

$$\Delta V=|V_{charge}-V_{discharge}|$$

***S1.2.8 Li||LFP pouch cells assembly and tests***

The preparation for the LFP cathode is same to described above with the average weight loading of LFP is approximately 4.6 mg∙cm^-2^. The thickness of Li anode is around 50 μm, thus the calculated N/P ratio is approximately 13.2. The fabrication process of Li||LFP pouch cells (3 × 3 cm²) were as follows: firstly, Li foils were used as the anodes, the aluminum foils loading with LFP were the cathodes, LLZTO-PMA were used as the electrolytes and segregated cathode and anode. Next, [ultrasonic](https://www.sciencedirect.com/topics/physics-and-astronomy/ultrasonics) [spot welding](https://www.sciencedirect.com/topics/materials-science/spot-welding) were employed to attach [aluminum](https://www.sciencedirect.com/topics/chemistry/aluminum) and nickel plates to aluminum collectors and Li anodes, ensuring a secure connection to the battery cycler. Finally, the pouch cells were then encapsulated in a specialized aluminum-plastic film with vacuum encapsulation. The fabricated Li||LFP pouch cells were tested to power a light-emitting diode at folding, penetration, and cutting conditions.

**S1.2.9 Li||NCM811 coin cells assembly and tests**

Similar to LFP electrode, NCM811 was mixed with carbon black and PVDF with weight ratio of 94:3:3 in NMP. The slurry was coated onto aluminum foil with an average weight of approximately 4.5 mg·cm^-2^, followed by vacuum drying and cell assembling like Li||LFP batteries. The cycling performance of Li||NCM811 were evaluated using the NEWARE battery testing system at 20 °C, with a charge-discharge rate of 0.5 C within a voltage range of 2.8 to 4.3 V (*vs.* Li/Li^+^)

**S2 Theoretic calculation section**

All the calculations were performed in the framework of the density functional theory with the projector augmented plane-wave method, as implemented in the Vienna ab initio simulation package. The generalized gradient approximation proposed by Perdew, Burke, and Ernzerhof was selected for the exchange-correlation potential. The cut-off energy for plane wave was set to 500 eV. The energy criterion was set to 10^−5^ eV in iterative solution of the Kohn-Sham equation. The Brillouin zone integration was performed using a 5x5x5 k-mesh. All the structures were relaxed until the residual forces on the atoms have declined to less than 0.01 eV/Å.

The energy barriers of Li atom diffusion on LiF, Li_3_N, and Li_3_N/LiF were simulated through the single point calculation with five images linearly generated between the initial and final states.

**S3 Supplementary Figures and Tables**


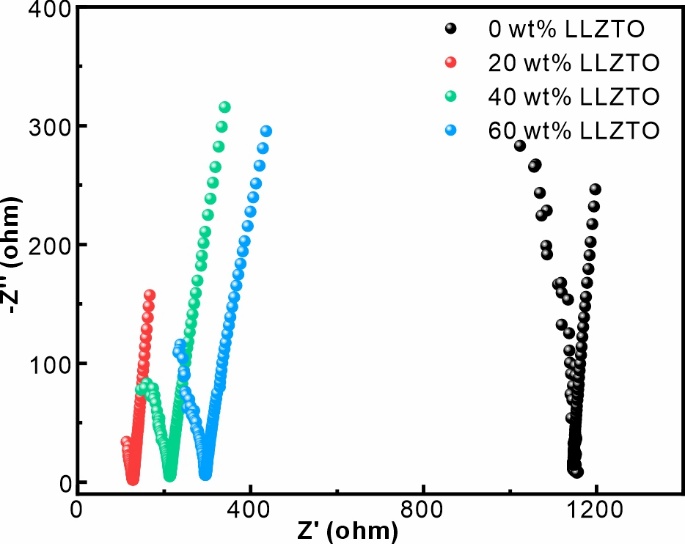


**Fig. S1** Nyquist plots of LLZTO-PMA with LLZTO contents of 0, 20, 40, and 60 wt%


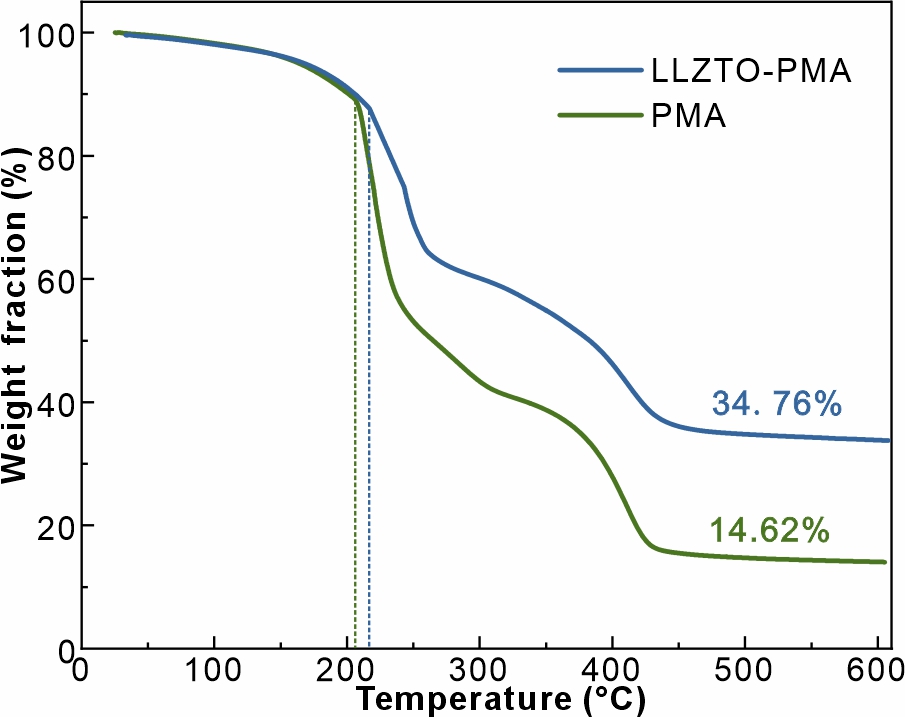


**Fig. S2** Thermogravimetric analysis curves of LLZTO-PMA and PMA electrolytes


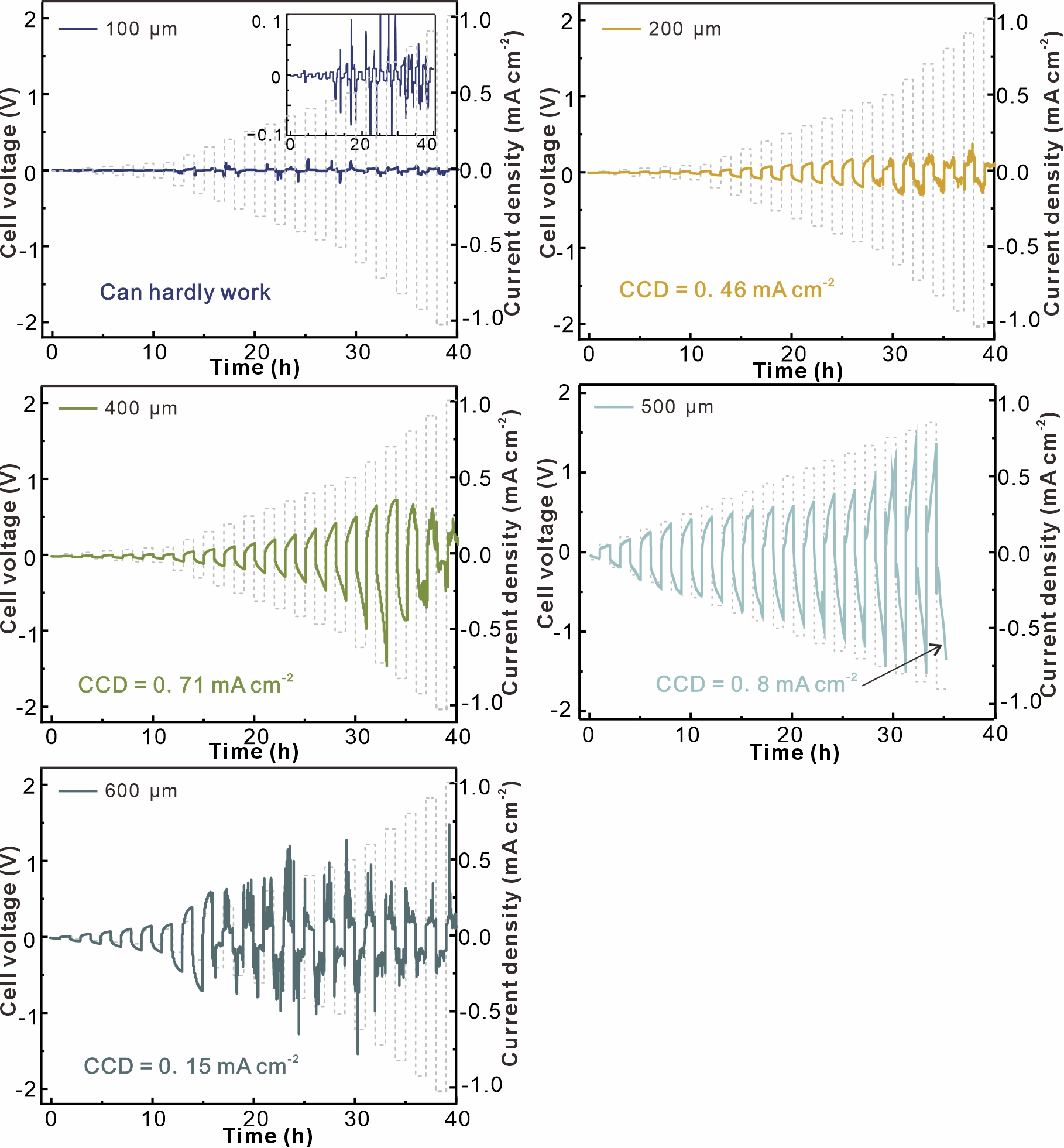


**Fig. S3** Critical current density of LLZTO-PMA electrolytes with different thickness


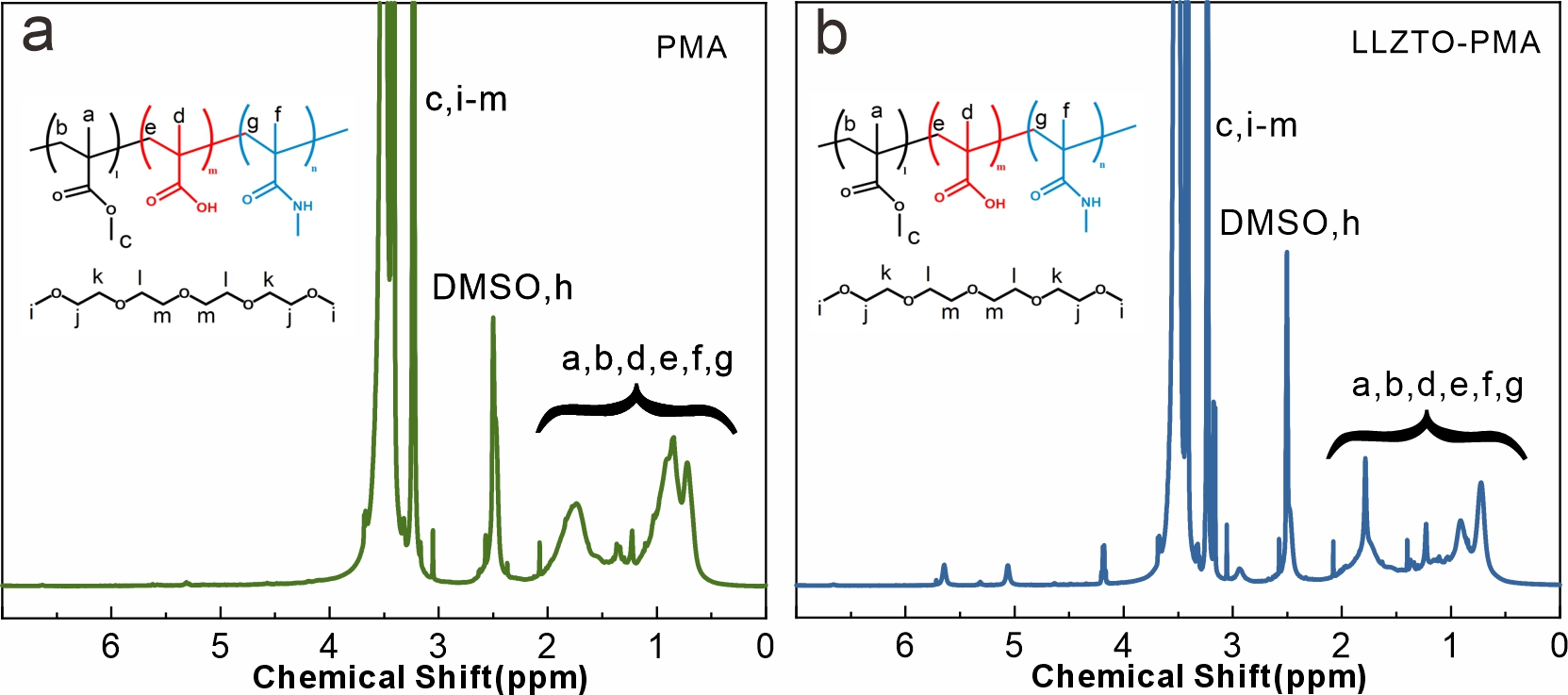


**Fig. S4** ^1^H-NMR spectroscopies of (**a**) PMA and (**b**) LLZTO-PMA


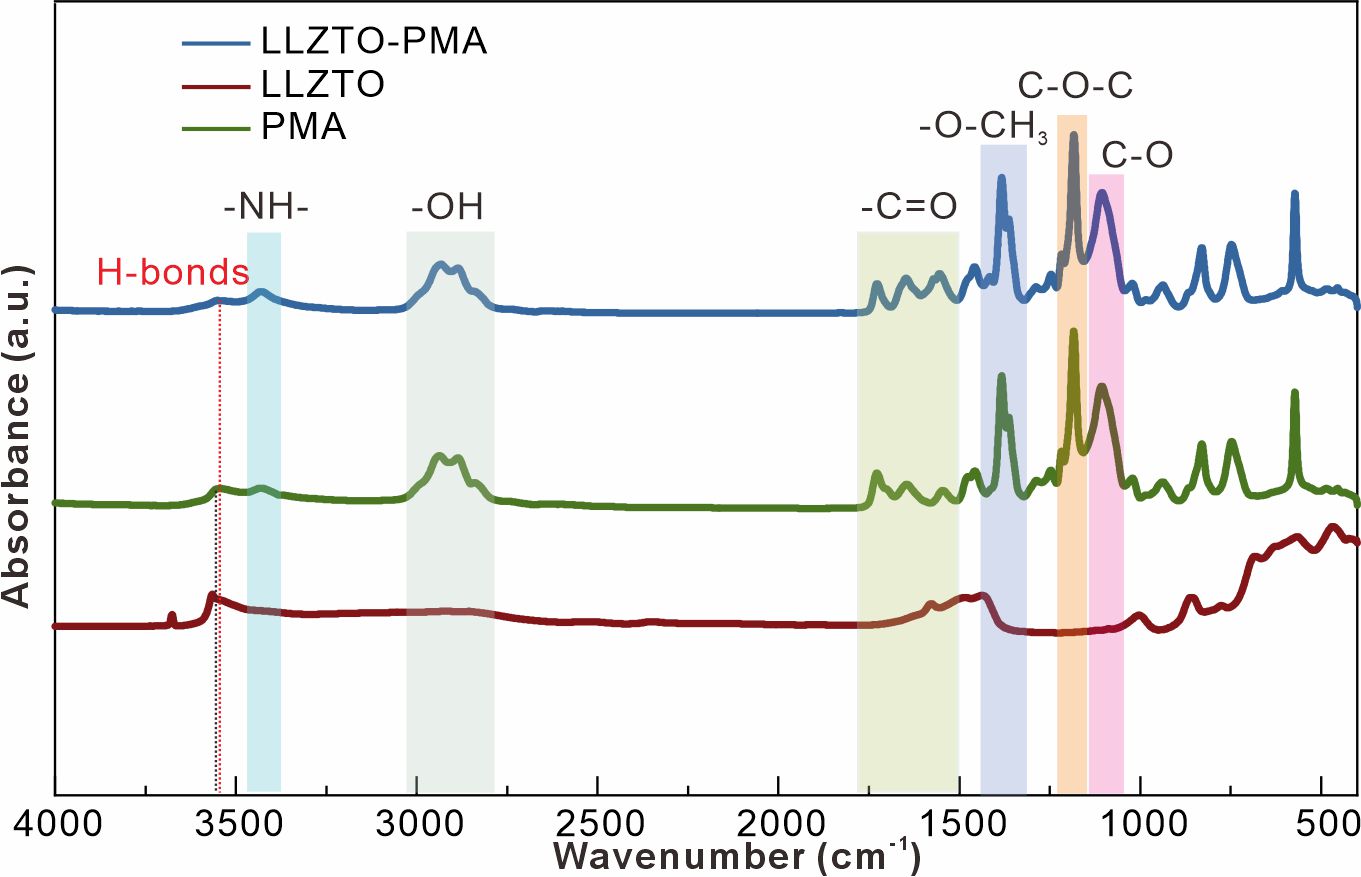


**Fig. S5** FTIR spectra of LLZO, PMA, and LLZTO-PMA


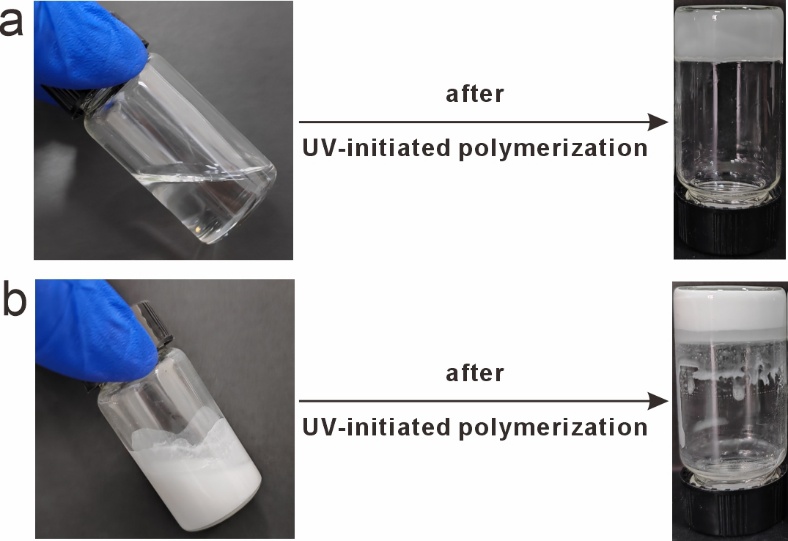


**Fig. S6** Optical photographs of (**a**) PMA and (**b**) LLZTO-PMA before and after copolymerization


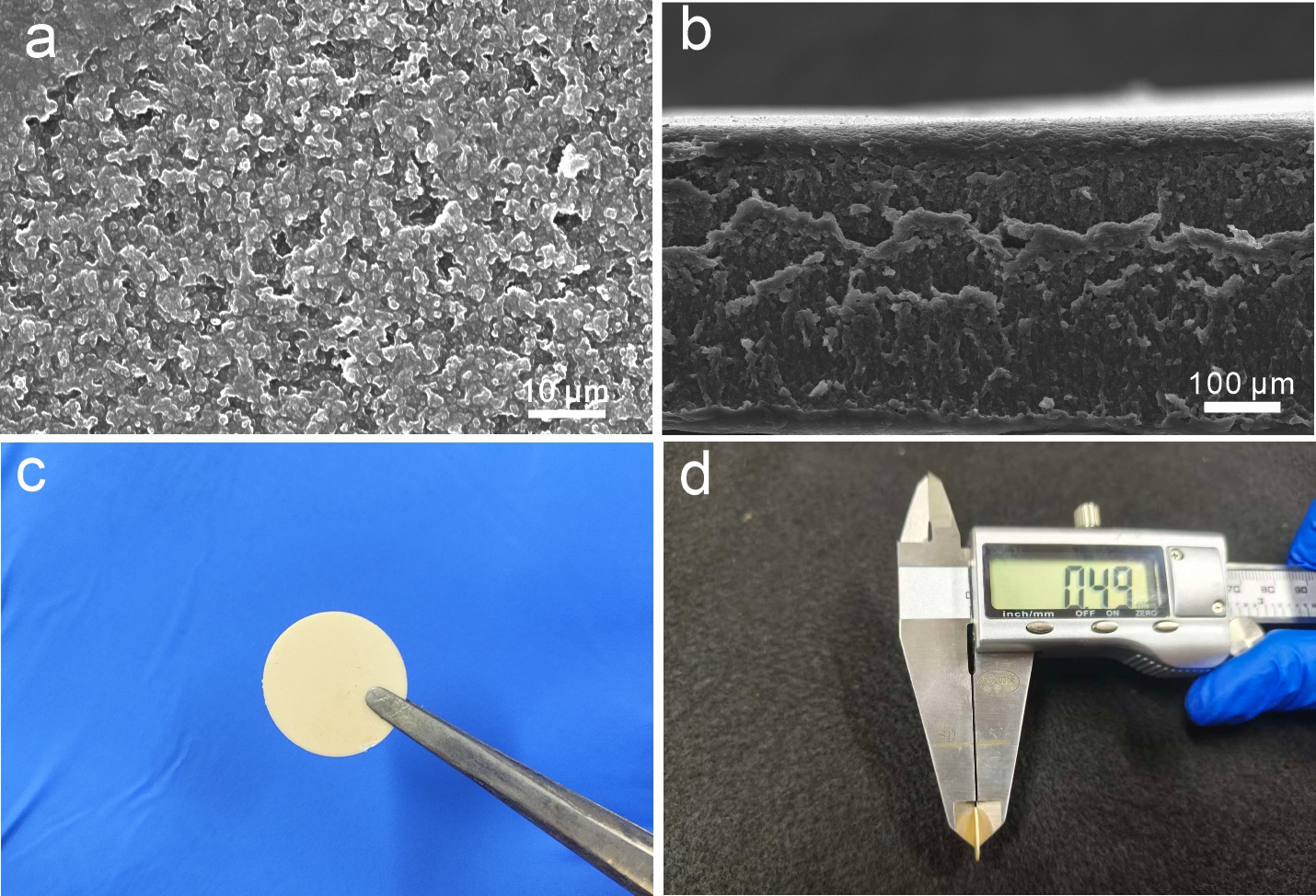


**Fig. S7** (**a**) SEM and (**b**) cross-section SEM images of PMA, and (**c, d**) optical photos of bare LLZTO


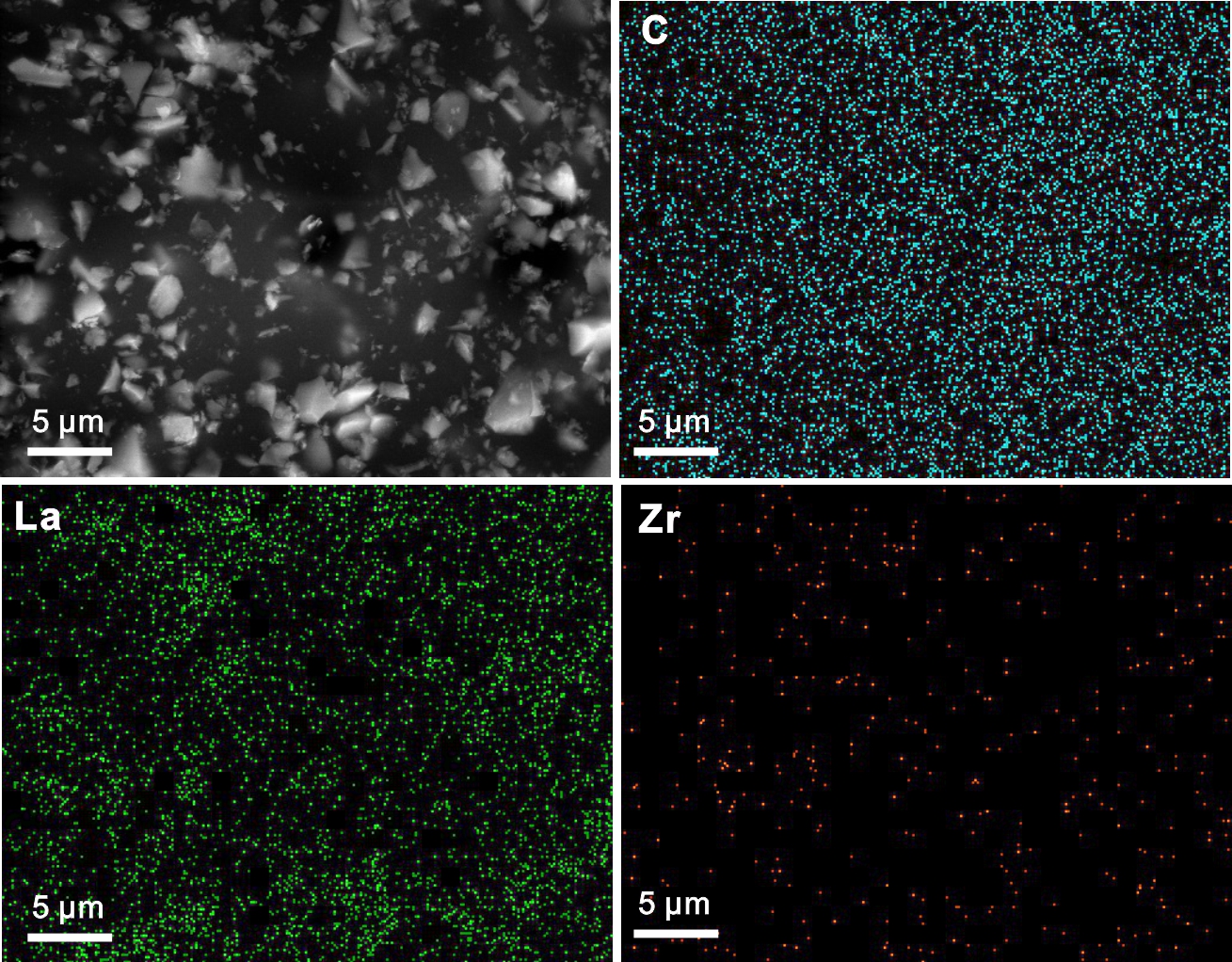


**Fig. S8.** C, La, and Zr elemental mapping of LLZTO-PMA


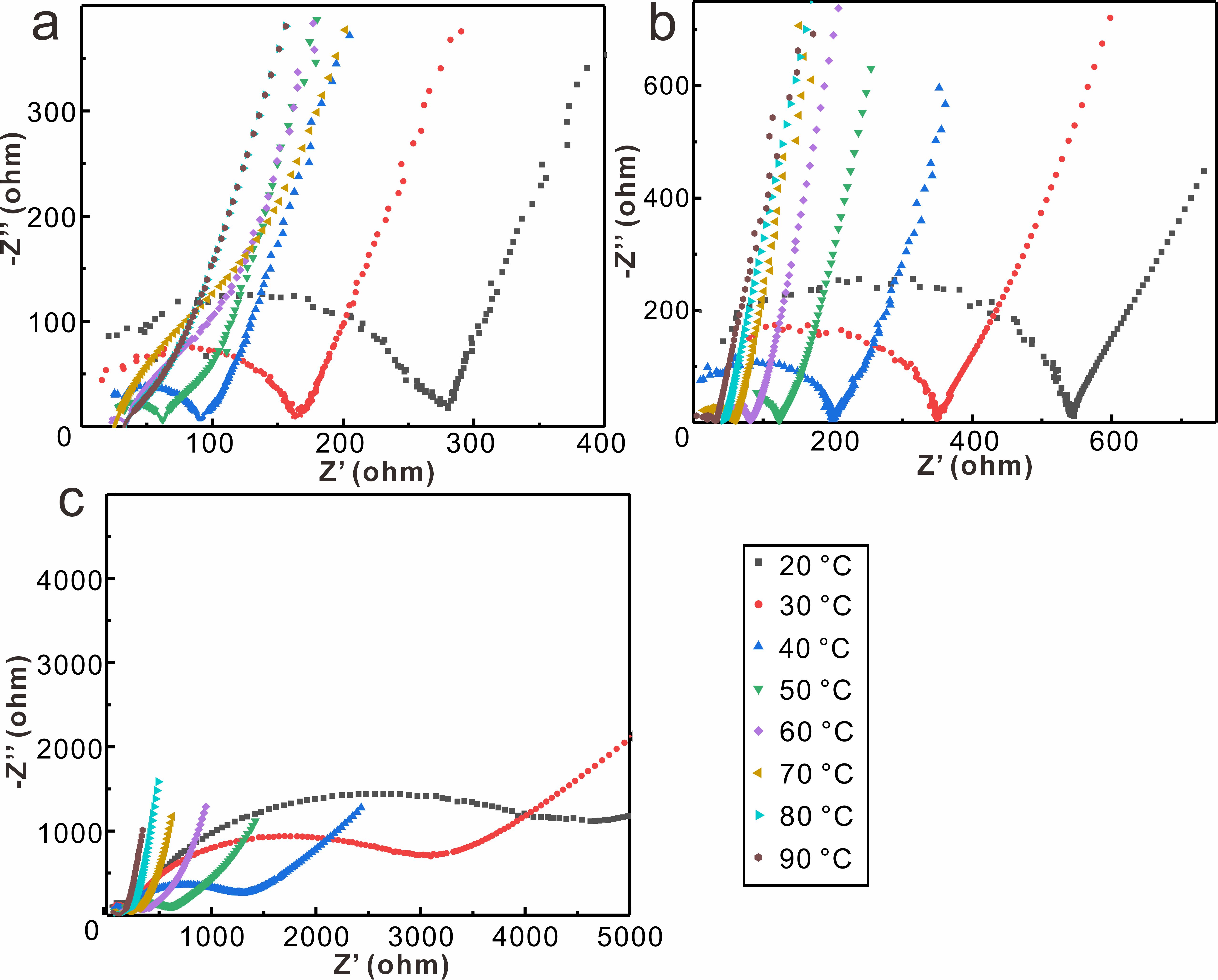


**Fig. S9** Nyquist plots of (**a**) LLZTO-PMA, (**b**) PMA, and (**c**) LLZTO electrolytes from 20 to 90 °C


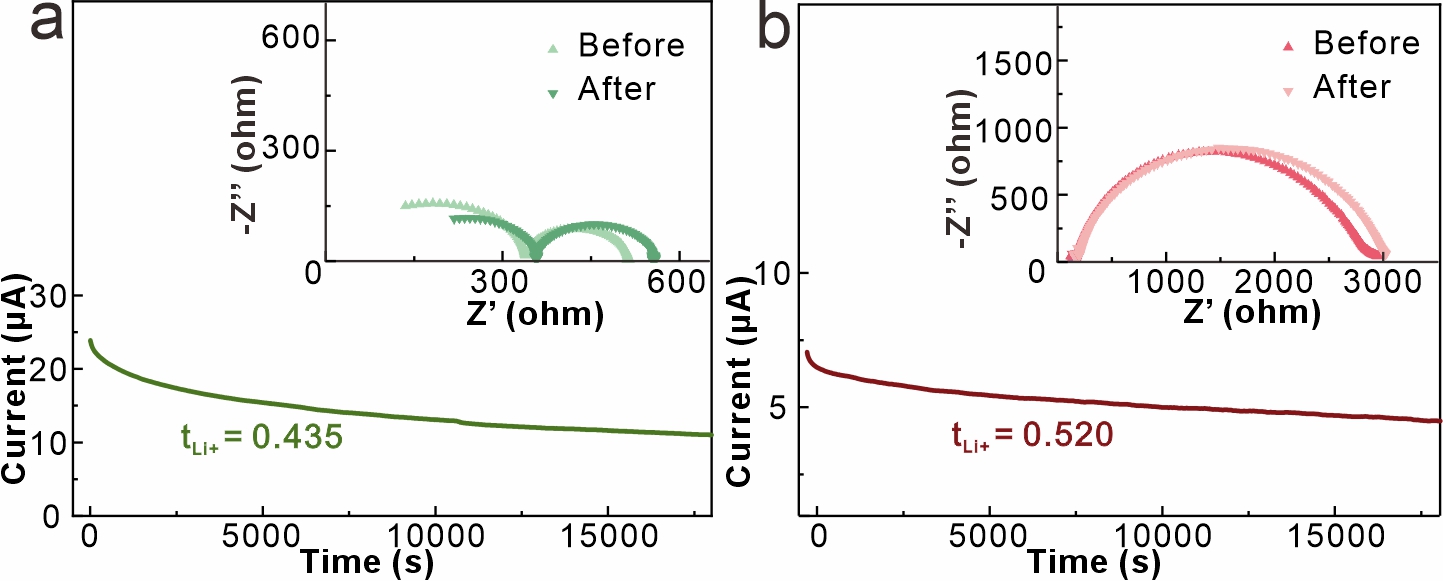


**Fig. S10** Chronoamperometry curves and AC impedance spectra before and after polarization of (**a**) Li|PMA|Li cells and (**b**) Li|LLZTO|Li cells


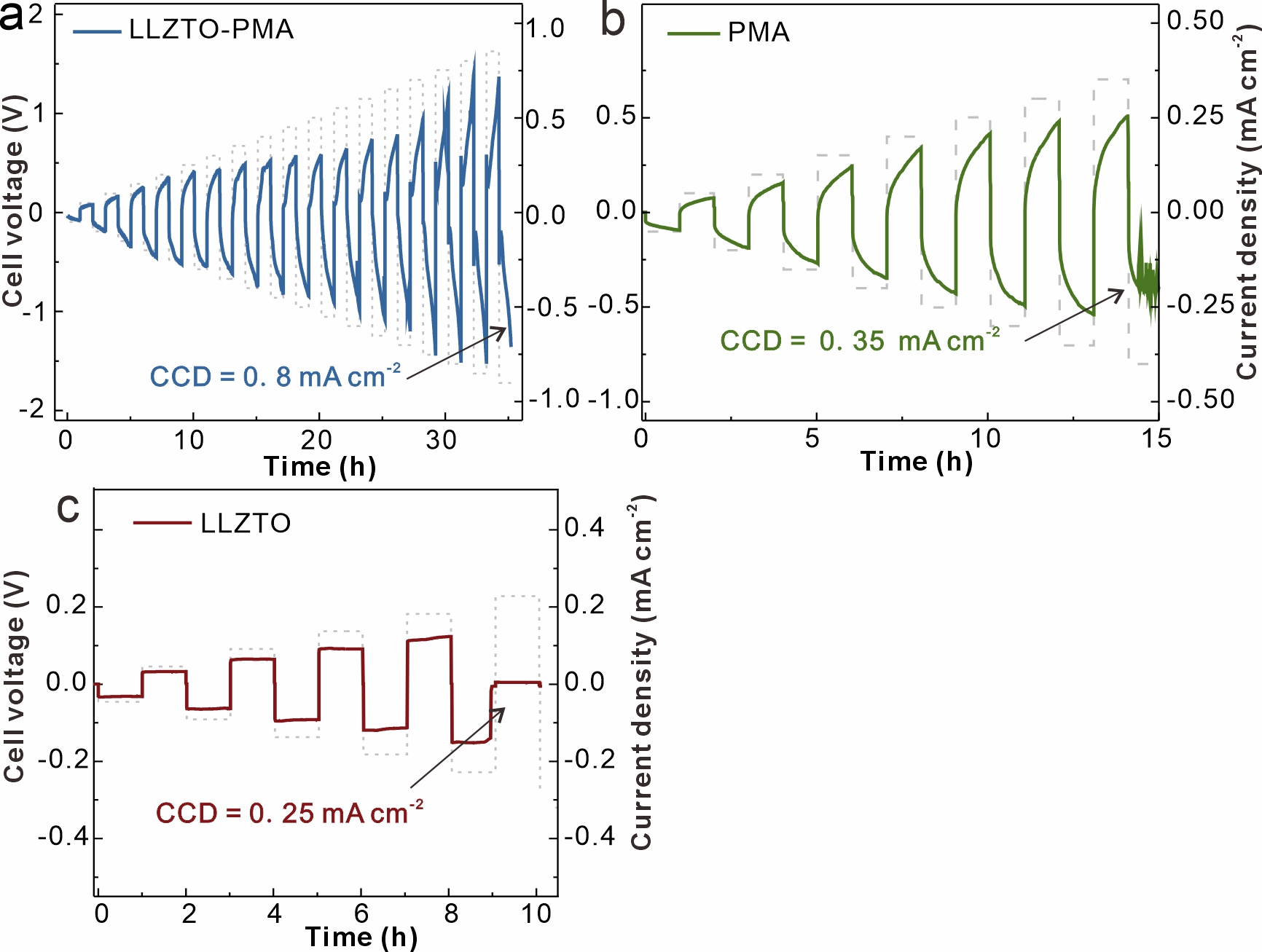


**Fig. S11** CCD measurements of Li||Li cells at 20 °C with (**a**) LLZTO-PMA, (**b**) PMA and (**c**) LLZTO electrolytes at increasing current densities ranging from 0.05 to 1 mA cm^−2^, with a stepwise increment of 0.05 mA cm^−2^


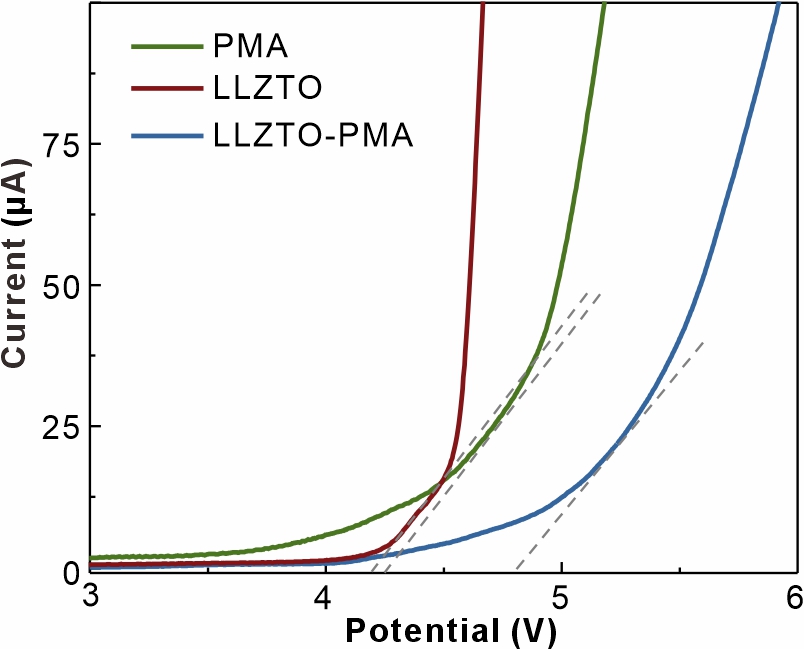


**Fig. S12** Liner sweep voltammetry plots of LLZTO-PMA, PMA and LLZTO electrolytes at 20 °C


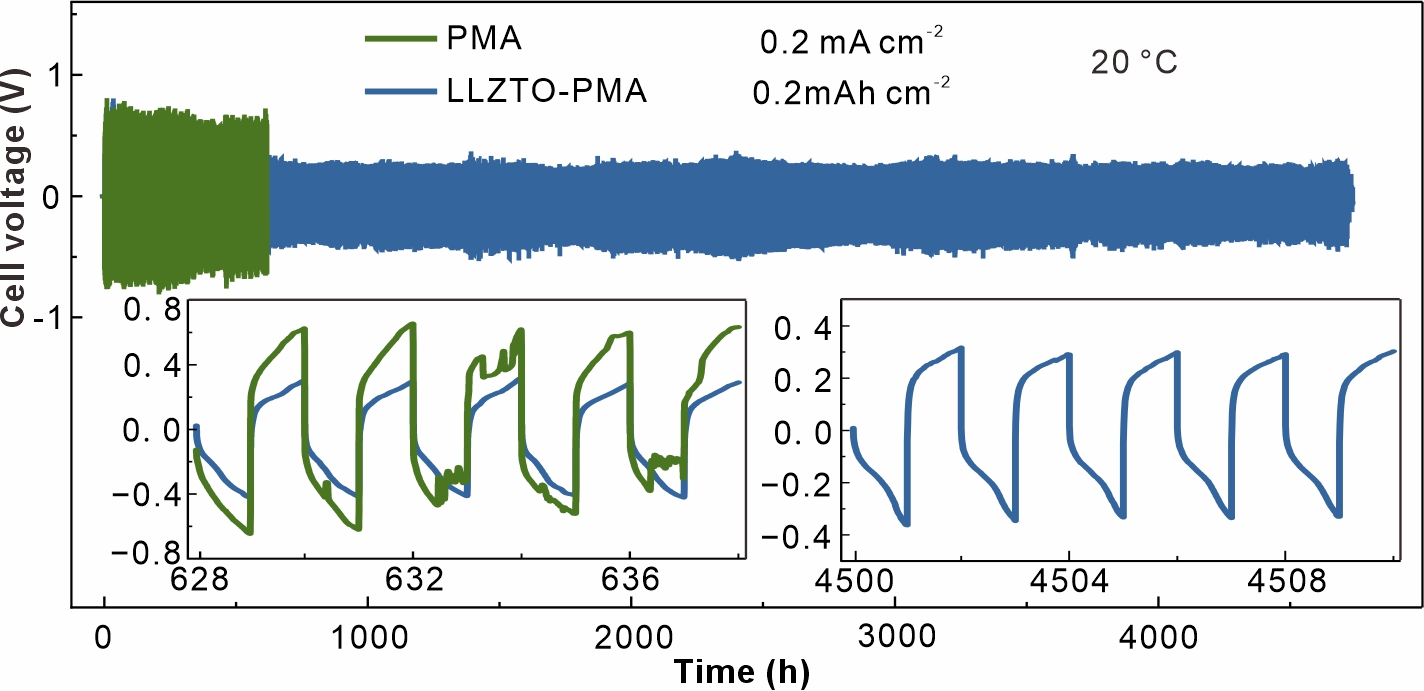


**Fig. S13** Galvanostatic Li plating/stripping of Li||Li cells at 20 °C with PMA and LLZTO-PMA electrolytes at 0.2 mA cm^−2^/0.2 mAh cm^−2^


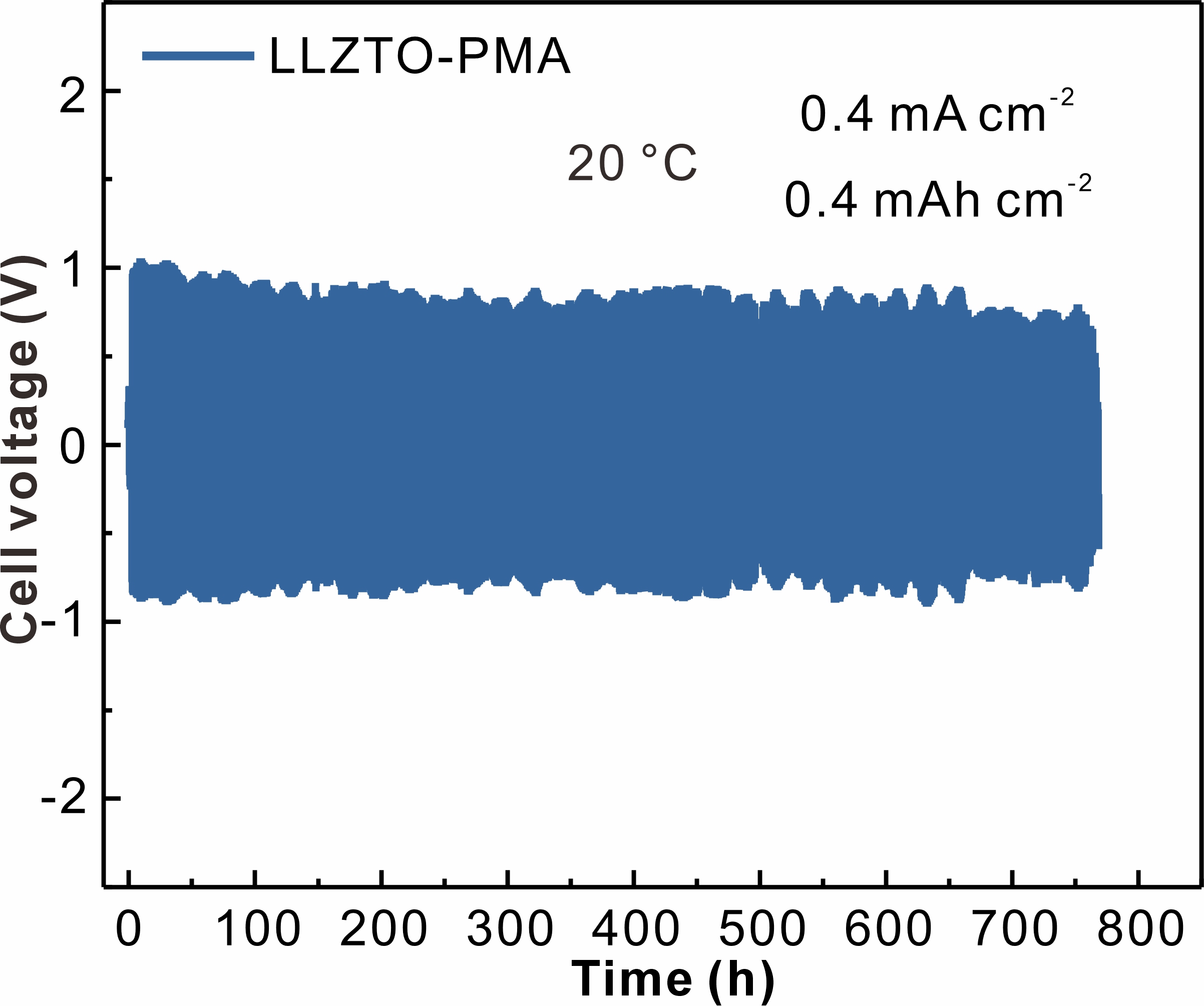


**Fig. S14** Galvanostatic Li plating/stripping of Li||Li cell at 20 °C with LLZTO-PMA electrolyte at 0.4 mA cm^−2^/0.4 mAh cm^−2^


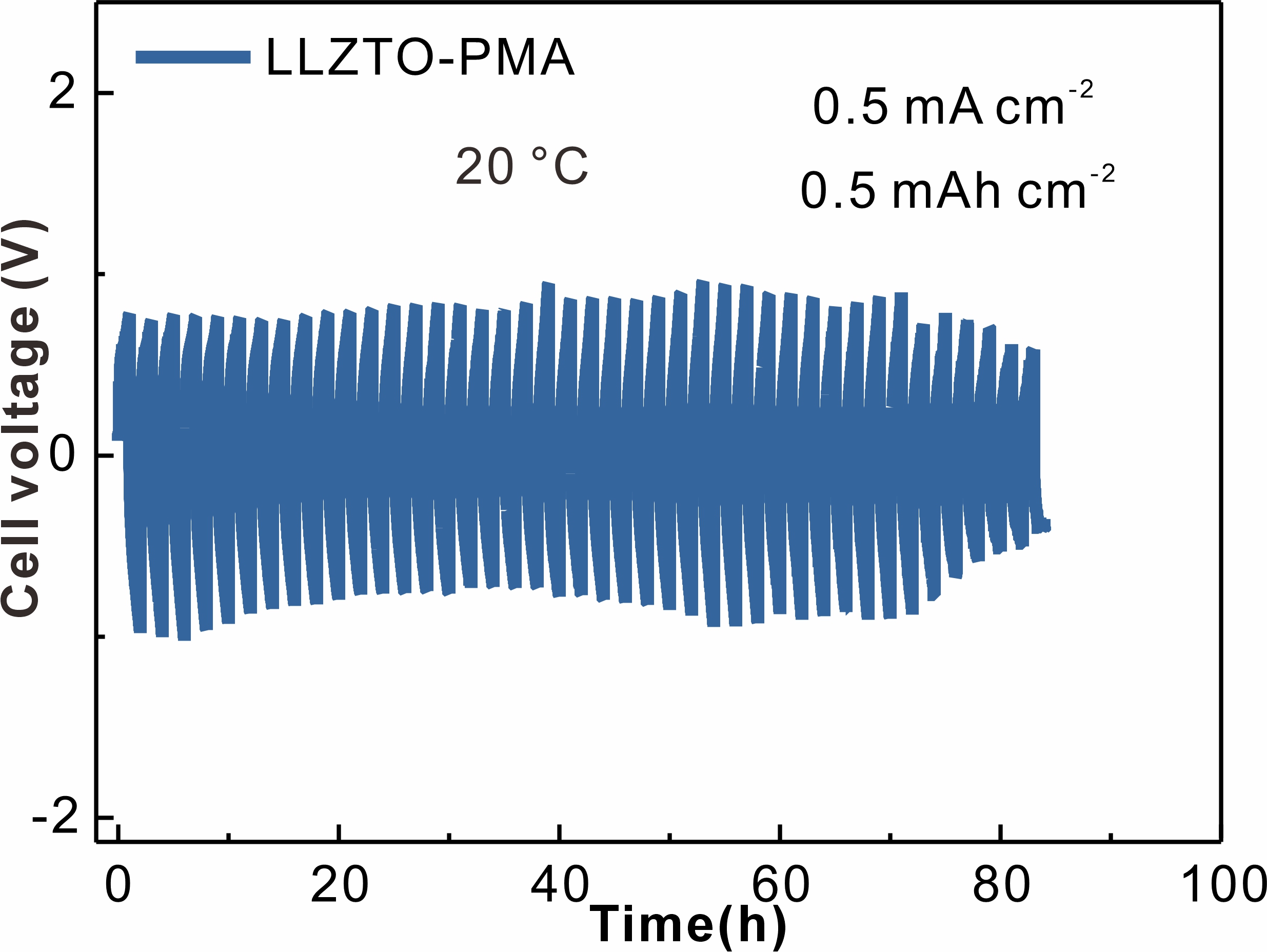


**Fig. S15** Galvanostatic Li plating/stripping of Li||Li cell at 20 °C with LLZTO-PMA electrolyte at 0.5 mA cm^−2^/0.5 mAh cm^−2^


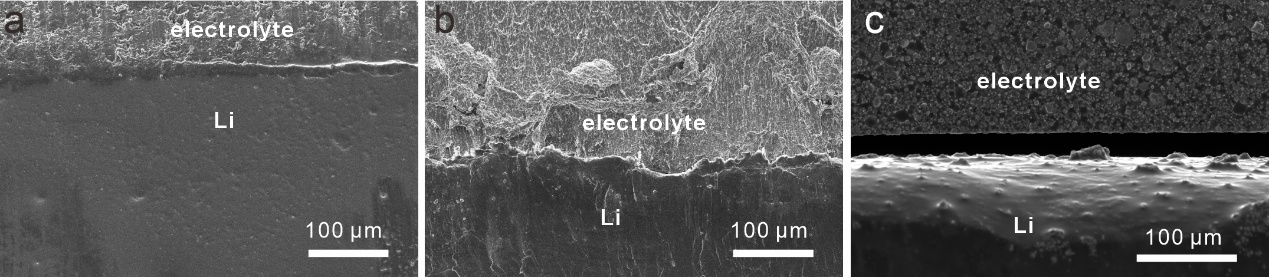


**Fig. S16** Cross-sectional SEM images of Li-electrolyte in symmetric cells with (**a**) LLZTO-PMA, (**b**) PMA after 100 cycles, and (**c**) LLZTO electrolytes after 50 cycles with 0.1 mA cm^-2^ at 20 °C


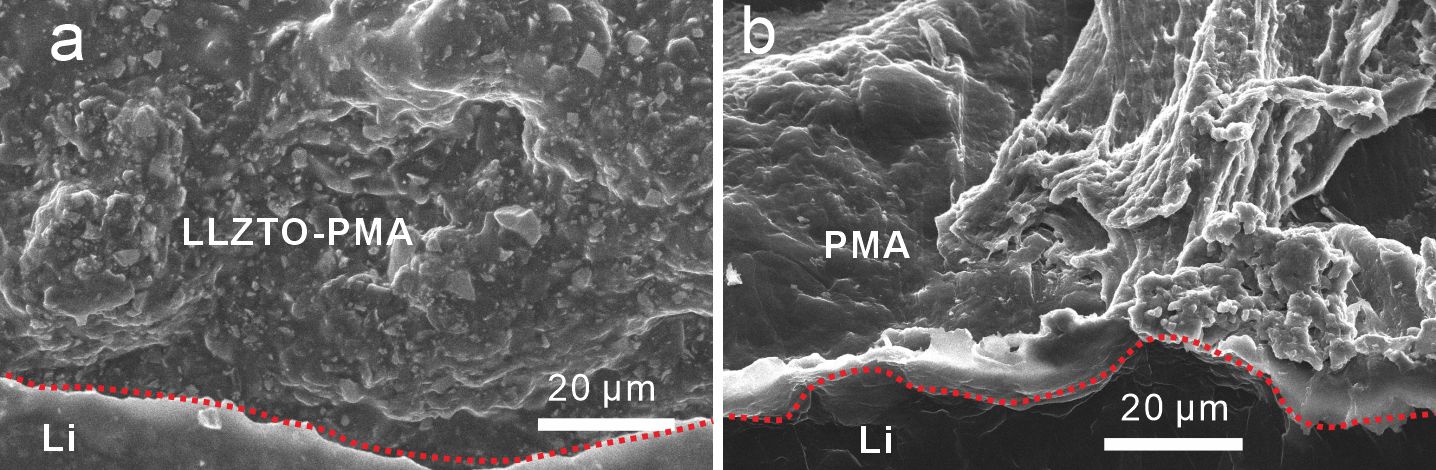


**Fig. S17** Cross-sectional SEM images of Li-electrolyte in symmetric cells with (**a**) LLZTO-PMA and (**b**) PMA electrolytes after 200 cycles with 0.1 mA cm^-2^ at 20 °C


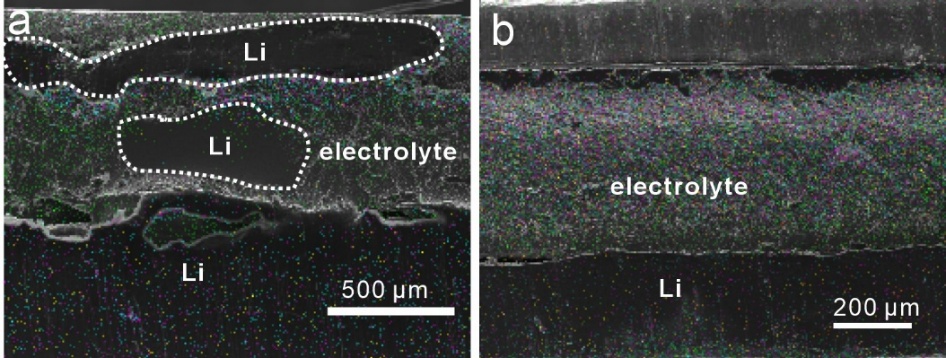


**Fig. S18** Cross-sectional SEM images and the corresponding EDX mapping of Li-electrolyte in symmetric cells with (**a**) PMA and (**b**) LLZTO-PMA electrolytes after 300 cycles with 0.1 mA cm^-2^ at 20 °C


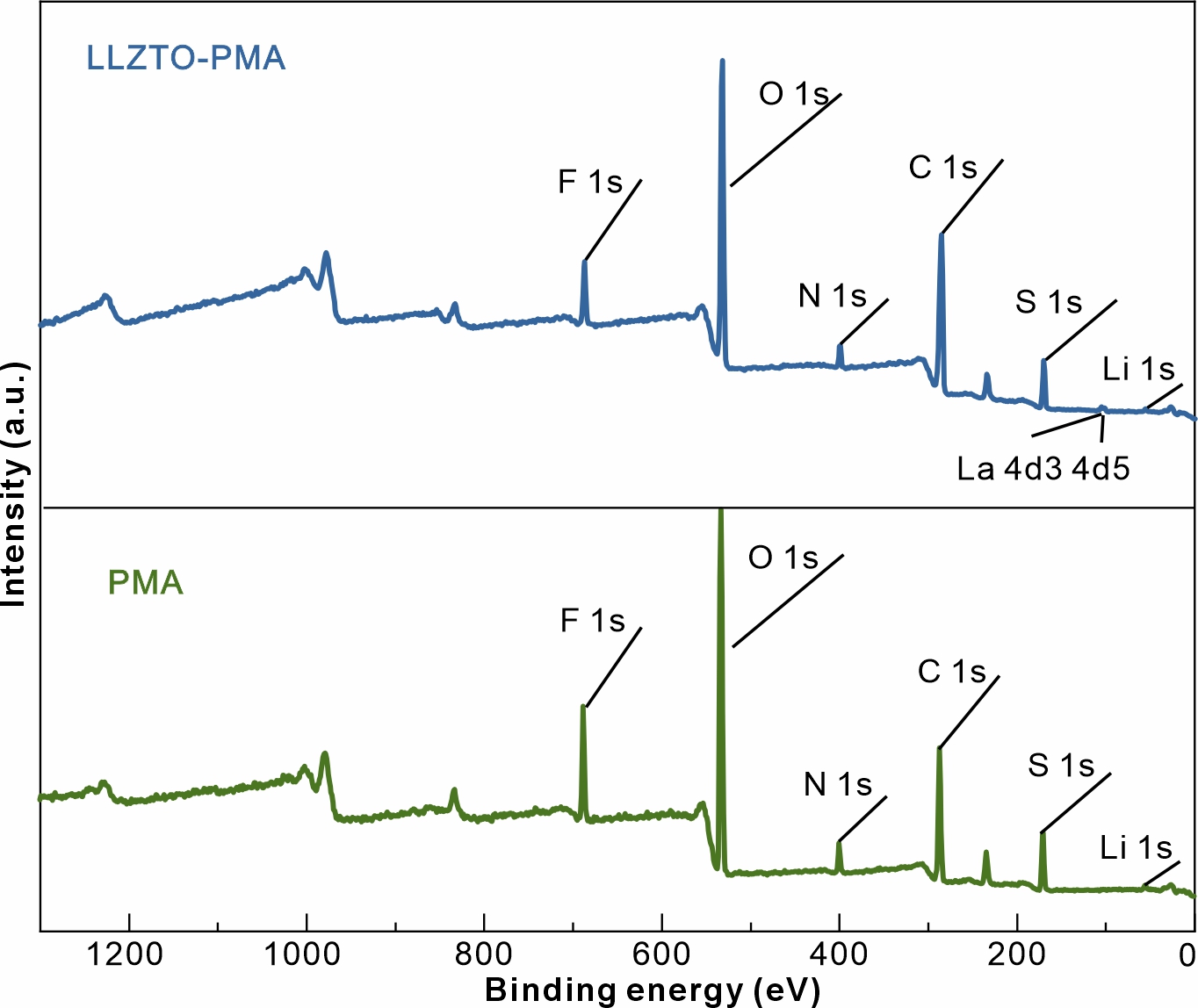


**Fig. S19** XPS survey of Li anode surface with PMA and LLZTO-PMA electrolytes based symmetric cells after 10 cycles at 0.1 mA cm^−2^


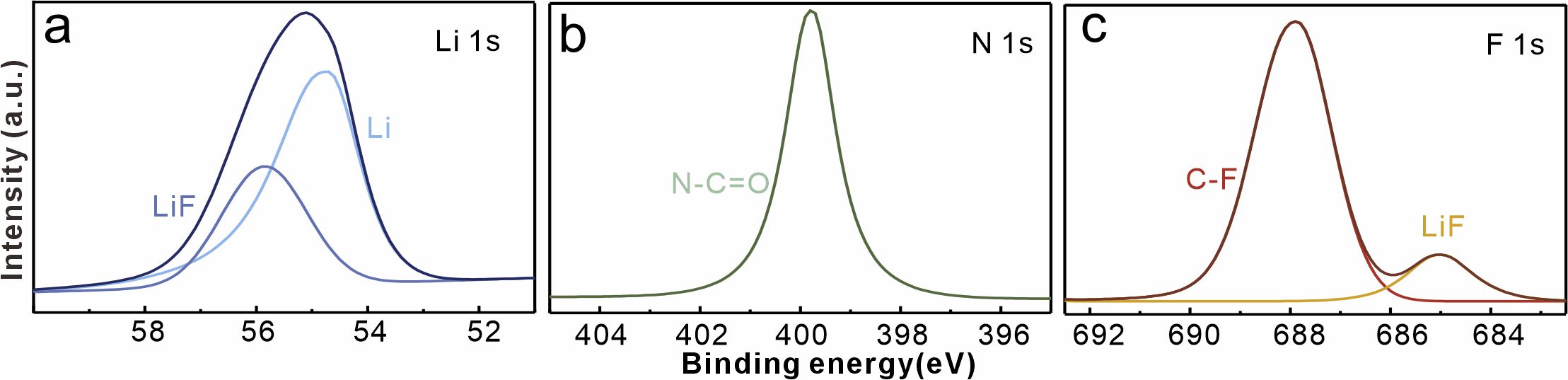


**Fig. S20** High-resolution XPS spectra of (**a**) Li 1s (**b**) N 1s, and (**c**) F 1s on Li surface with PMA electrolyte based symmetric cells after 10 cycles at 0.1 mA cm^−2^


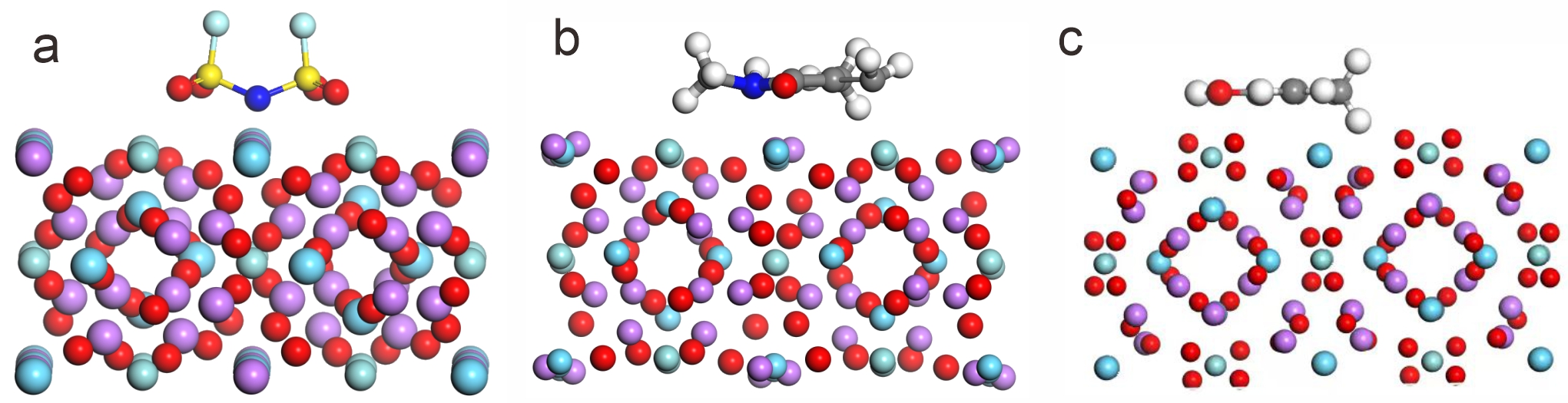


**Fig. S21** Optimized molecular configuration diagram of (**a**) FSI^-^ anion, (**b**) NMMA and (**c**) MAA after incorporation with LLZTO


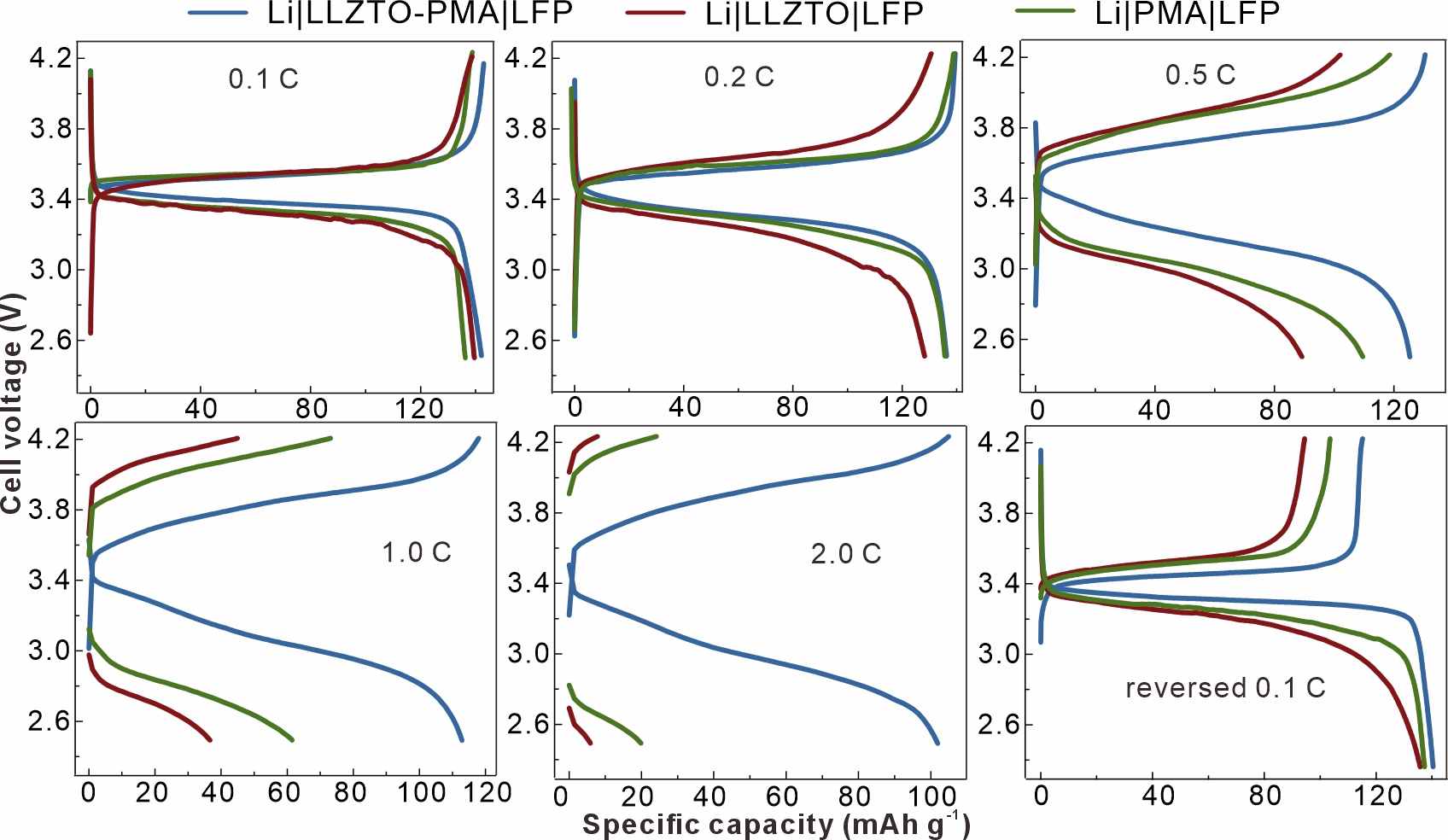


**Fig. S22** Voltage profile comparisons of Li||LFP batteries with LLZTO, PMA, and LLZTO-PMA electrolytes at different rates under 20 °C


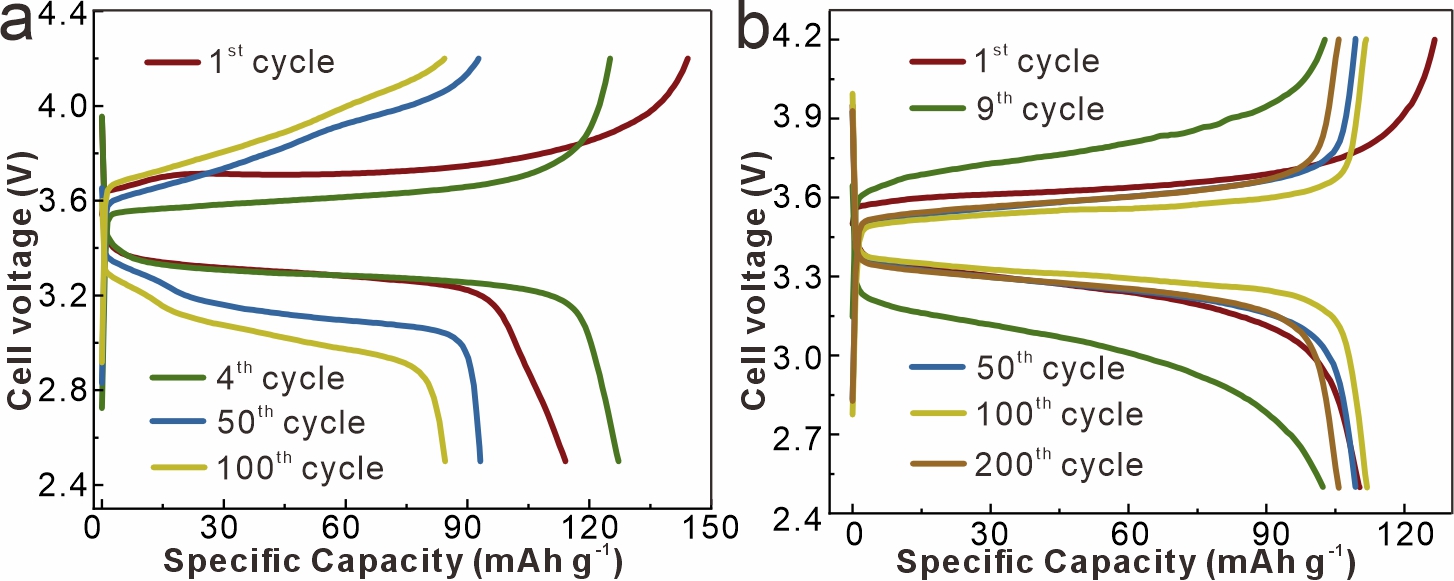


**Fig. S23** Voltage profiles of (**a**) Li|PMA|LFP and (**b**) Li|LLZTO|LFP batteries at 0.2 C with different cycles


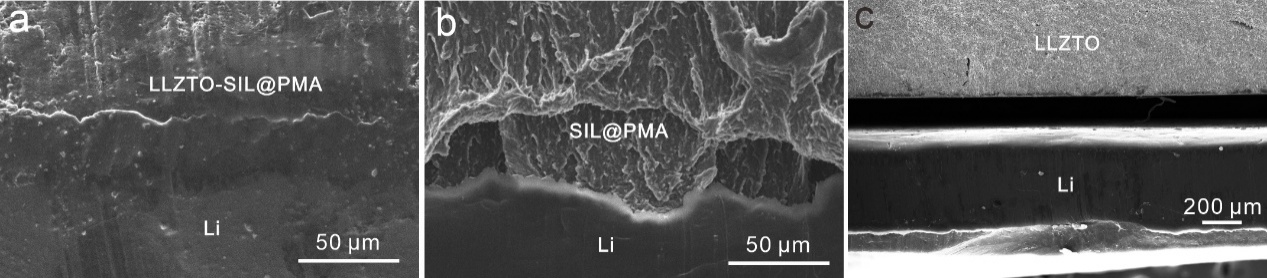


**Fig. S24** Cross-section SEM images of electrolyte-Li anodes in (**a**) Li|LLZTO-PMA|LFP, (**b**) Li|PMA|LFP, and (**c**) Li|LLZTO|LFP batteries after 100 cycles at 0.2 C


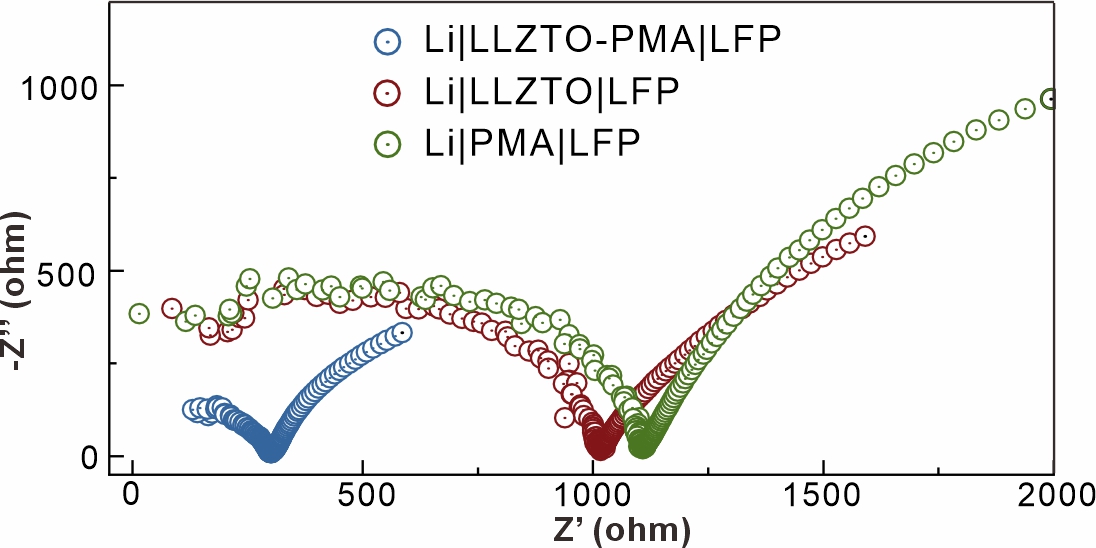


**Fig. S25** Nyquist plots of Li||LFP batteries with LLZTO-PMA, PMA, and LLZTO electrolytes after 100 cycles at 0.2 C


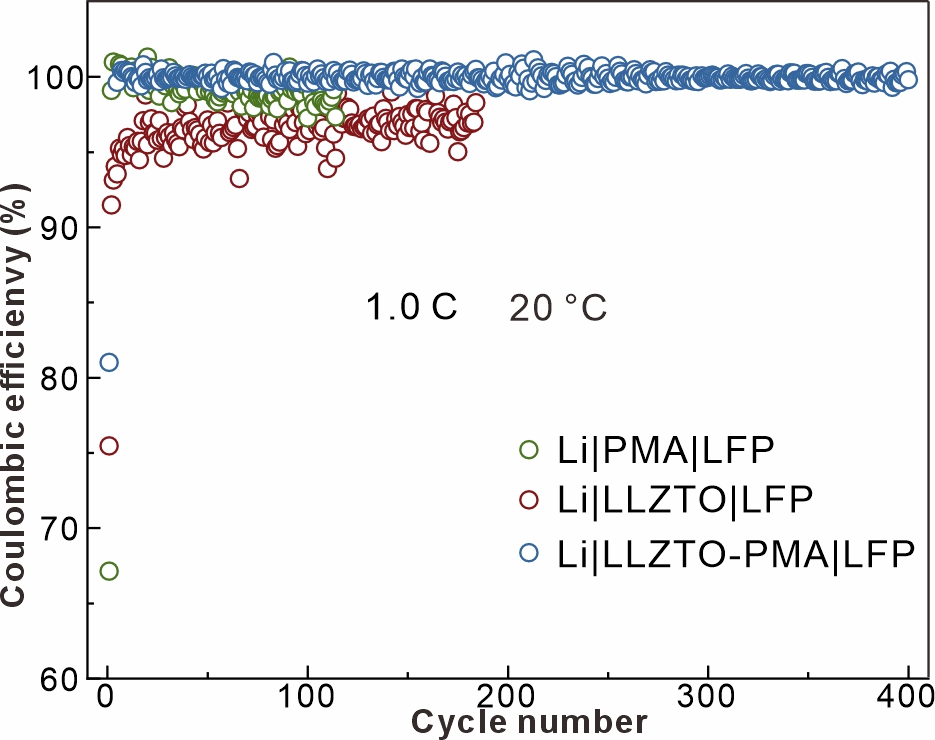


**Fig. S26** Coulombic efficiency of Li|LLZTO-PMA|LFP, Li|PMA|LFP and Li|LLZTO|LFP batteries at 1.0 C


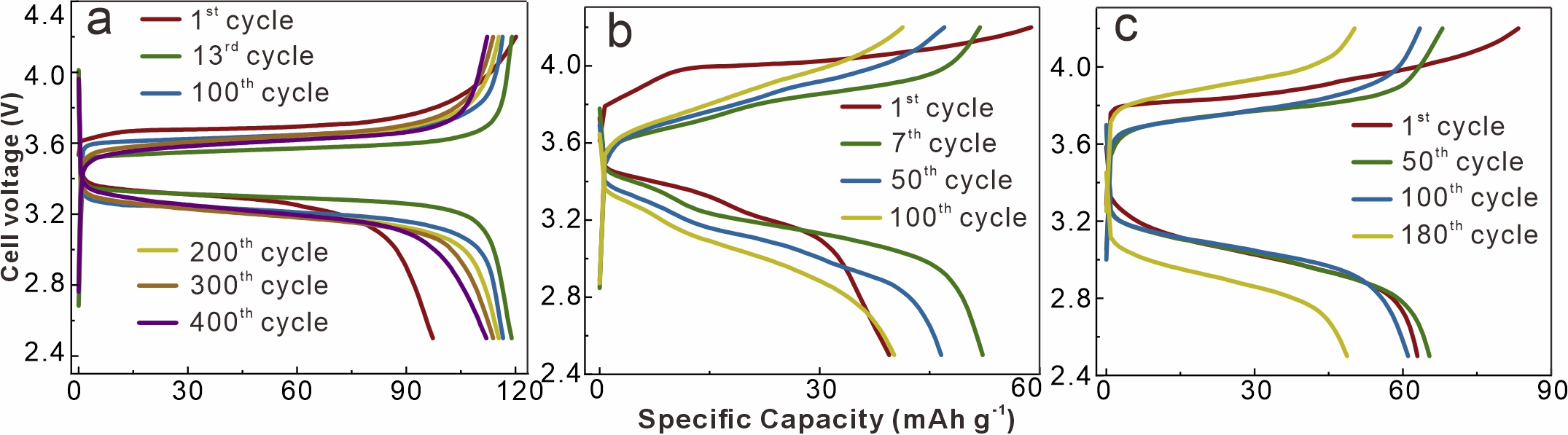


**Fig. S27** Voltage profiles of (**a**) Li|LLZTO-PMA|LFP, (**b**) Li|PMA|LFP and (**c**) Li|LLZTO|LFP batteries at 1.0 C with different cycles


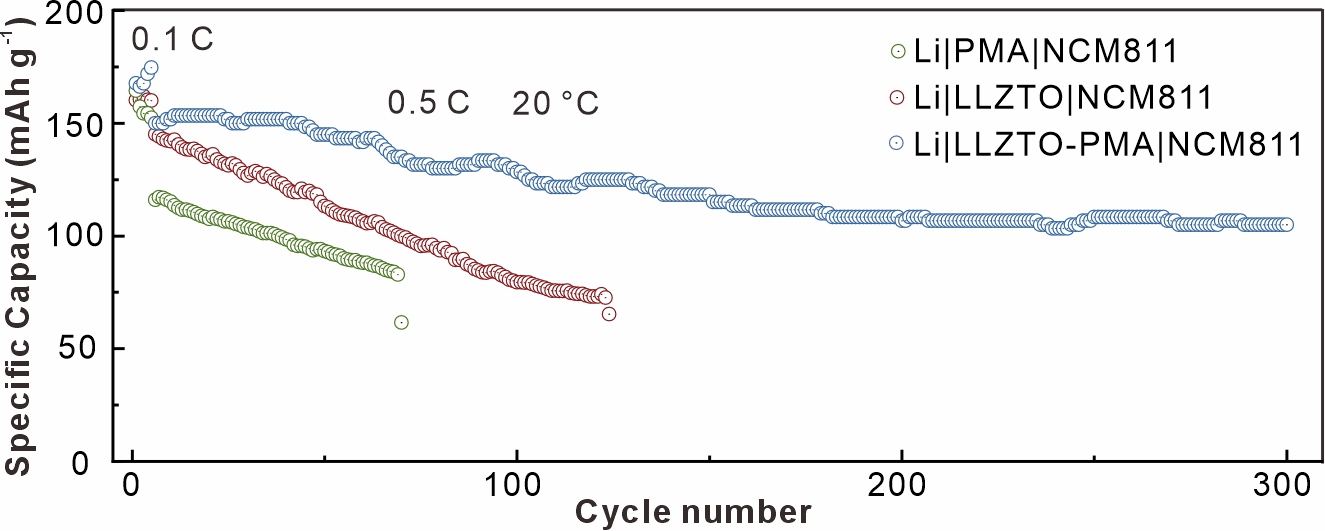


**Fig. S28** Cycling performance of Li||NCM811 batteries with LZLTO, PMA, and LLZTO-PMA electrolytes at 0.5 C

**Table S1** Calculated interfacial impedances of LLZTO-PMA, PMA, and LLZTO electrolyte based cells after different cycles at 20 °C and 0.1 mA cm^−2^

|  | 1^st^ cycle | 10^th^ cycle | 100^th^ cycle | 200^th^ cycle | 300^th^ cycle |
| --- | --- | --- | --- | --- | --- |
| LLZTO-PMA (Ω) | 472.2 | 354.5 | 365.3 | 392.2 | 415.4 |
| PMA (Ω) | 310.2 | 172.4 | 580.9 | 51.0 | 656.4 |
|  | **1^st^ cycle** | **10^th^ cycle** | **30^th^ cycle** | **50^th^ cycle** |  |
| LLZTO ((Ω)) | 882.7 | 615.8 | 748.1 | 1040.0 |  |

**Table S2** The comprehensive comparison of the LLZTO-PMA electrolyte with other similar systems

| LLZO-based composite electrolyte | Ionic conductivity  (mS cm^-1^) | Highest Rate | LFP-based full batteries performance | Refs. |
| --- | --- | --- | --- | --- |
| LLZTO@SALi/PEO | 0.12 (R.T.) | 2 C | 113.8 mA cm^-2^/ 1400 cycle/ 1 C/ R.T. | [S1] |
| LLZO NFs/PEO | 0.939 (60 °C) | 1 C | 109 mA cm^-2^/ 400 cycle/ 0.5 C/ 60 °C | [S2] |
| Ga-V-LLZO/PEO | 0.0498 (R.T.) | 5 C (60 °C) | 117.4 mA cm^-2^/ 300 cycle/ 0.5 C/ R.T. | [S3] |
| Ga-LLZO/PVDF-HFP | 0.729 (30 °C) | 1 C | 89.6 mA cm^-2^/ 280 cycle/ 1 C/ R.T. | [S4] |
| LLZO-PAN-PC | 0.229 (R.T.) | 2 C | 110 mA cm^-2^/ 300 cycle/ 0.5 C/ 60 °C | [S5] |
| LLZO/poly (cyclocarbonate-ether) | 0.717 (R.T.) | 1.5 C (40 °C) | 139.4 mA cm^-2^/ 550 cycle/ 0.5 C/ 40 °C | [S6] |
|  |  |  | 134.8 mA cm^-2^/ 400 cycle/ 1 C/ 40 °C |  |
| LLZTO/PAN/Ti_3_C_2_T_x_/PEO | 0.217 (30 °C) | 5 C (60 °C) | 100 mA cm^-2^/ 300 cycle/ 0.3 C/ 35 °C | [S7] |
|  |  |  | 160 mA cm^-2^/ 300 cycle/ 0.5 C/ 60 °C |  |
|  |  |  | 139 mA cm^-2^/ 300 cycle/ 1 C/ 60 °C |  |
| 3D LLZO‐PAN | 0.29 (R.T.) | \ | \ | [S8] |
| LLZTO/ionogel | 0.24 (R.T.) | 2 C | 117.6 mA cm^-2^/ 500 cycle/ 1 C/ R.T. | [S9] |
| LLZO@ZIF-67-PEO | 0.109 (R.T.) | \ | 139.7 mA cm^-2^/ 100 cycle/ 0.1 C/ 60 °C | [S10] |
| Al-Ta-LLZO/PVDF-HFP | 0.268 (R.T.) | 0.5 C | 136.5 mA cm^-2^/ 100 cycle/ 0.2 C/ R.T. | [S11] |
| LLZO-PDOL | 0.5 (35 °C) | \ | 150 mA cm^-2^/ 50 cycle/ 20 mA g^-1^/ R.T. | [S12] |
| 3D-LLZTO-PVDF | 0.167 (R.T.) | 2 C | 115.3 mA cm^-2^/ 450 cycle/ 1 C/ R.T. | [S13] |
| LLZO-PEO | 0.21 (R.T.) | 2 C | 160 mA cm^-2^/ 200 cycle/ 0.1 C/ R.T. | [S14] |
| LLZTO-PMA | 0.266 (20 °C) | 2 C | 138.2 mA cm^-2^/ 610 cycle/ 0.2 C/ 20 °C | This work |
|  | 0.44 (30 °C) |  | 112 mA cm^-2^/ 400 cycle/ 1 C/ 20 °C |  |
|  | 1.72 (60 °C) |  |  |  |

**Supplementary References**

1. X. Zhan, X. Pang, F. Mao, J. Lin, M. Li et al., Interfacial reconstruction unlocks inherent ionic conductivity of Li-La-Zr-Ta-O garnet in organic polymer electrolyte for durable room-temperature all-solid-state batteries. Adv. Energy Mater. **14**(42), 2402509 (2024). <https://doi.org/10.1002/aenm.202402509>
2. Y. Teng, H. Liu, Q. Wang, Y. He, Y. Hua et al., In-doped Li_7_La_3_Zr_2_O_12_ nanofibers enhances electrochemical properties and conductivity of PEO-based composite electrolyte in all-solid-state lithium battery. J. Energy Storage **76**, 109784 (2024). <https://doi.org/10.1016/j.est.2023.109784>
3. Y. Li, X. Gao, W. Wang, J. Shi, H. Wang, Ga, V dual-doped LLZO modified PEO matrix composite solid-state electrolyte for fast Li^+^ conduction and ultra-stable all-solid-state lithium batteries. J. Power Sources **629**, 236027 (2025). <https://doi.org/10.1016/j.jpowsour.2024.236027>
4. S. Paengson, P. Pilasuta, K. Yuzou, M. Mao, D. Mori et al., High-performance PVDF-HFP/LLZO-Ga polymer electrolytes with improved ionic conductivity and stability for quasi-solid lithium metal batteries. J. Mater. Sci. **60**(28), 11973–11993 (2025). <https://doi.org/10.1007/s10853-025-11143-7>
5. Y. Ji, H. Du, K. Wang, J. Ma, W. Yang et al., Self-purification and surface-coordination dual-polymer/Li_6.4_La_3_Zr_1.4_Ta_0.6_O_12_ interphase achieves Li^+^-transport-enhanced composite solid-state electrolyte. J. Power Sources **646**, 237289 (2025). <https://doi.org/10.1016/j.jpowsour.2025.237289>
6. X. Zheng, D. Xu, N. Fu, Z. Yang, Ultrathin poly(cyclocarbonate-ether)-based composite electrolyte reinforced with high-strength functional skeleton. J. Energy Chem. **81**, 603–612 (2023). <https://doi.org/10.1016/j.jechem.2023.03.006>
7. H. Xu, S. Liu, Z. Li, F. Ding, J. Liu et al., Synergistic effect of Ti_3_C_2_T*_x_* MXene/PAN nanofiber and LLZTO particles on high-performance PEO-based solid electrolyte for lithium metal battery. J. Colloid Interface Sci. **668**, 634–645 (2024). <https://doi.org/10.1016/j.jcis.2024.04.201>
8. X. Zhao, C. Wang, X. Fan, Y. Li, D. Li et al., Addressing the interface issues of all-solid-state lithium batteries by ultra-thin composite solid-state electrolyte combined with the integrated preparation technology. InfoMat **7**(8), e70012 (2025). <https://doi.org/10.1002/inf2.70012>
9. W. Lin, H. Yuan, C. Tian, M. Song, T. Huang et al., Inorganic fillers tailored Li^+^ solvation sheath for stable lithium metal batteries. Energy Storage Mater. **70**, 103472 (2024). <https://doi.org/10.1016/j.ensm.2024.103472>
10. X. Yang, Y. Liu, D. Li, J. Dong, F. Chai et al., Preparation of high ionic conductivity composite solid electrolyte with *in-situ* synthesis of LLZO@ZiF-67. Electrochim. Acta **431**, 141155 (2022). <https://doi.org/10.1016/j.electacta.2022.141155>
11. J. Zou, X. Gao, X. Zhou, J. Yang, J. Tang et al., Al and Ta Co-doped LLZO as active filler with enhanced Li+ conductivity for PVDF-HFP composite solid-state electrolyte. Nanotechnology **34**(15), 155402 (2023). <https://doi.org/10.1088/1361-6528/acb3cb>
12. L. Chen, X. Huang, R. Ma, W. Xiang, J. Ma et al., A nanocrystal garnet skeleton-derived high-performance composite solid-state electrolyte membrane. Energy Storage Mater. **65**, 103140 (2024). <https://doi.org/10.1016/j.ensm.2023.103140>
13. B. Wei, Y. Li, W. Lin, J. Yu, D. Chen, A wrapped and infiltrated ∼20-μm-thick 3D ceramic framework composite enables fast Li^+^ diffusion and interfacial compatibility for lithium-metal batteries. Compos. Part B Eng. **272**, 111192 (2024). <https://doi.org/10.1016/j.compositesb.2024.111192>
14. H. Zhang, X. An, Y. Yang, Y. Long, S. Nie et al., Vertical aligned solid-state electrolyte templated by nanostructured “upright” cellulose film layers for advanced cell performance. EcoMat **5**(4), e12317 (2023). <https://doi.org/10.1002/eom2.12317>
